# Identification of 1, 2, 4-Triazine and Its Derivatives Against Lanosterol 14-Demethylase (CYP51) Property of *Candida albicans:* Influence on the Development of New Antifungal Therapeutic Strategies

**DOI:** 10.3389/fmedt.2022.845322

**Published:** 2022-03-28

**Authors:** Abhishek Kumar Verma, Aarfah Majid, Md. Shahadat Hossain, SK. Faisal Ahmed, Mohammad Ashid, Ali Asger Bhojiya, Sudhir K. Upadhyay, Naveen Kumar Vishvakarma, Mudassir Alam

**Affiliations:** ^1^Department of Biosciences, Manipal University, Jaipur, India; ^2^Department of Chemistry, Faculty of Science and Technology, Mewar University, Chittorgarh, India; ^3^Department of Biotechnology and Genetic Engineering, Noakhali Science and Technology University, Noakhali, Bangladesh; ^4^Department of Science, U.S. Ostwal Science, Arts & Commerce College, Chittorgarh, India; ^5^Department of Environmental Science, V.B.S. Purvanchal University, Jaunpur, India; ^6^Department of Biotechnology, Guru Ghasidas Vishwavidyalaya, Bilaspur, India; ^7^Department of Zoology, Aligarh Muslim University, Aligarh, India

**Keywords:** 1, 2, 4-triazine, Lanosterol 14-demethylase (CYP51), drug resistance, molecular docking, molecular dynamic simulation

## Abstract

This research aims to find out whether the 1, 2, 4-triazine and its derivatives have antifungal effects and can protect humans from infection with *Candida albicans*. Molecular docking and molecular dynamic simulation are widely used in modern drug design to target a particular protein with a ligand. We are interested in using molecular docking and molecular dynamics modeling to investigate the interaction between the derivatives of 1, 2, 4-triazine with enzyme Lanosterol 14-demethylase (CYP51) of *Candida albicans*. The inhibition of *Candida albicans* CYP51 is the main goal of our research. The 1, 2, 4-triazine and its derivatives have been docked to the CYP51 enzyme, which is involved in *Candida albicans* Multidrug Drug Resistance (MDR). Autodock tools were used to identify the binding affinities of molecules against the target proteins. Compared to conventional fluconazole, the molecular docking results indicated that each drug has a high binding affinity for CYP51 proteins and forms unbound interactions and hydrogen bonds with their active residues and surrounding allosteric residues. The docking contacts were made using a 10 ns MD simulation with nine molecules. RMSD, RMSF, hydrogen bonds, and the Rg all confirm these conclusions. In addition, these compounds were expected to have a favorable pharmacological profile and low toxicity. The compounds are being offered as scaffolds for the development of new antifungal drugs and as candidates for future *in vitro* testing.

## Introduction

*Candida* species, the most common of which are *C. albicans*, cause most of the fungal infections in humans. The presence of *Candida albicans* is noteworthy. *Candida albicans* is often found as part of the normal microbiota in the human intestine but can cause life-threatening infections in immunocompromised people such as HIV patients (1). *Candida* species can also cause bloodstream infections, and with the increase in candida infections in recent years, multi-drug resistance (MDR) has become a major public health problem (2, 3). MDR is a property in which cells become resistant to multiple chemotherapy drugs that are not chemically related at the same time. Drug efflux pumps from the ATP-Binding Cassette (ABC) or Major Facilitator Superfamily (MFS) families play an important role in establishing and maintaining drug tolerance. Because of their rapid expulsion, induced overexpression of genes that produce these transporter proteins is unable to enable cells to maintain dangerous levels of the drug, rendering it unusable (3–6).

CYP51 belongs to the cytochrome P450 superfamily of monooxygenases, which catalyzes the oxidative removal of the 14-methyl group (C-32) from lanosterol to give 14, 15-desaturated intermediates in ergosterol biosynthesis in fungi produce kingdoms, CYP51 is an important enzyme in sterol biosynthesis, which fulfills metabolic functions such as membrane permeability, membrane fluidity, enzyme activity, cell shape, and progression of the cell cycle (7–9). This enzyme occurs in all eukaryotes (including humans) and because azoles also interact with other cytochrome P450-dependent enzymes (CYP3A4), specific inhibition of the enzyme is decisive for a higher therapeutic index (10–13), the hydrophilic H-bond region, and thus the small hydrophobic gap formed. The affinity of azole antifungals for CYP51 is determined not only by the coordinative binding of the nitrogen of the azole ring to the heme iron within the active side (N-4 of triazole and N-3 of imidazole) but also by the affinity of the N-l substituent to the apoprotein, which is part of the remainder of the azole antifungal agent fits into the hydrophobic groove of CYP51 in the same way as Lanosterol (14–20).

1, 2, 4-Triazine and its derivatives can be a common core structural system found in a variety of physiologically active chemicals, including antifungal and antibacterial activity, and are also known to move pharmacologically (21).

This work aimed to use a computer-aided drug discovery strategy to find 1, 2, 4-triazine and its derivatives that have the potential to inhibit the protein *Candida albicans* Lanosterol 14-demethylase (CYP51). The binding of ligand to protein is accounted by performing molecular docking, and the stability of the complex formed by docking of the protein and ligand is verified by performing the molecular dynamic simulations. In this present paper, an *in silico* study done with 1, 2, 4-triazine and its derivatives as an inhibitor will help in establishing the strong candidature of 1, 2, 4-triazine and its derivatives as a potential drug target against fungal infection.

## Materials and Methodology

### Protein Preparation

**Figure 1:**
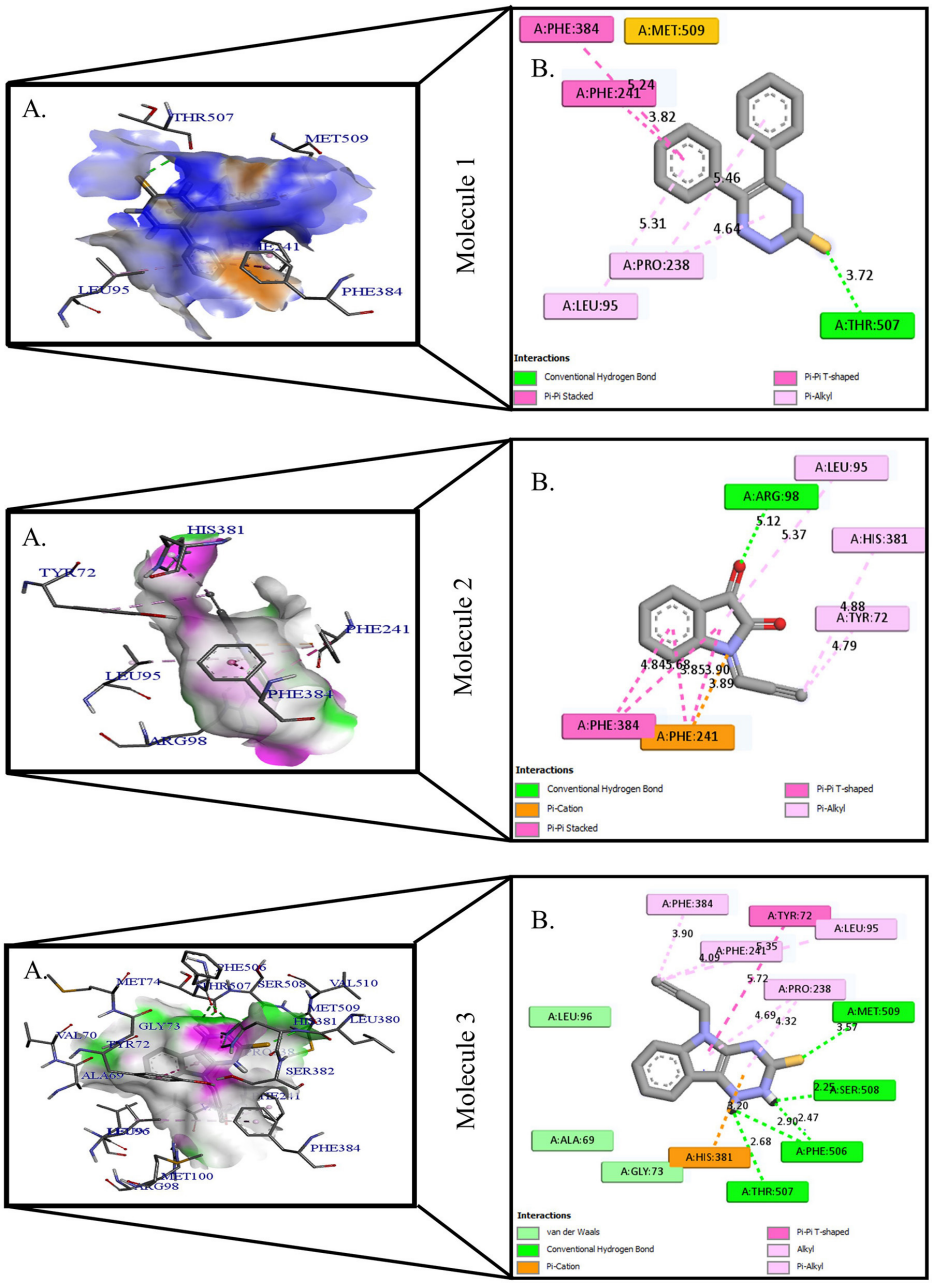
Protein-ligand presentation of CYP51 with molecules 1, 2 and 3. **(A)** 3D receptor-ligand presentation, and **(B)** 2D receptor-ligand interactions.

The protein database (https://www.rcsb.org/) was used to obtain the structure of Lanosterol 14-Demethylase (CYP51) (PDB ID: 4LXJ) (22). PDB is a database containing information about experimental proteins and nucleic acid structures. Water molecules were removed with PyMOL (Figure 1). PyMOL is an open-source software program that can be used to create molecular graphics (23).

### Ligand Preparation

The 1, 2, 4-triazine and its derivatives were selected for their potential pharmacological and therapeutic benefits (Supplementary Figure 1) (21). Docking research was conducted against fluconazole, a controlled drug that may be a potent CYP51 inhibitor and is commonly given to mycoses. The structures of the control drug fluconazole were retrieved from the PubChem database. The PubChem database contains data on chemical compounds, including their structure, formula, molecular weight, etc. (https://pubchem.ncbi.nlm.nih.gov/source/15751) (24). ChemDraw software was used to create all derived connections in the mol file. The structures were first called up in SDF format and then translated into PDB format using PyMOL, an open-source system program for molecular visualization (23).

### Binding Site Prediction

The Computed Atlas for Surface Topography of Proteins was used to identify the amino acids that are involved in the formation of active pockets (CASTp). CASTp could be a simple and helpful web-based tool for the identification of protein structures and site pockets (25). Determining the location is crucial for aligning the mesh box before docking.

### Molecular Docking

The molecular docking simulations were performed using Auto Dock (The Scripps Research Institute, La Jolla, CA, USA). It is widely used as open software for molecular docking and significantly increases the accuracy of binding mode predictions compared to Auto Dock 4 (26–29) and site residues of the proteins were estimated using the web-based CASTp tool. The size () of the lattice box in CYP51 was 88.63, 86.49, and 60.53, while the middle (x, y, and z) of the lattice box was 22.01, 14.21, and 19.82.

### Analysis and Visualization of Docking Results

The docked posture with the best negative score was selected as the best for the appropriate chemical and protein after the docking simulation. PyMOL Molecular Graphics System 2.0 (23) and Discovery Studio 4.5 (30) were used to display and score the best-docked posture to study unbound interactions.

### Molecular Dynamic Simulation

Molecular dynamics calculations were used to determine the physical motions of atoms and molecules in a protein-ligand-docked complex. The nine molecules were selected for a 10 ns MD simulation. The docked complexes were constructed using the steepest descent minimization module, the YASARA energy minimization module (YASARA Biosciences, GmbH), and the AMBER force field (Assisted Model Building with Energy Refinement) (31, 32) before proceeding with the MD simulation Force field, 298 K temperature, 1 bar pressure, Coulomb electrostatics with a cut-off of 7.86, 0.9% NaCl, solvent density 0.997, pH 7.0, 1-fs time steps, periodic boundaries, and all mobile atoms were used in the MD simulation (33, 34). The binding energy, the root mean square deviation (RMSD), the root mean square fluctuation (RMSF), the radius of gyration (Rg), and the total number of H-bonds were measured with MD determines simulations in the same way as in previous research (35–37).

### MMPBSA Calculations

Molecular mechanics / Poisson-Boltzmann surface (MMPBSA) (38) is one of the most frequently used methods for calculating the binding free energy of a protein-ligand combination. The stable region of the nine molecules with CYP51 complexes was used to construct a 10 ns MD trajectory for MM-PBSA calculations. The MMPBSA method of the YASARA simulator was used to measure the binding energy components (YASARA Biosciences, GmbH). The g_mmpbsa tool uses the following equation to calculate the binding energy of the protein-ligand complex.


$$\begin{array}{l} \text{Δ}{\text{G}}_{\text{Binding}}={\text{G}}_{\text{Complex}}-\left({\text{G}}_{\text{Protein}}+{\text{G}}_{\text{Ligand}}\right) \end{array}$$

Where G_Complex_ denotes the binding complex's total free energy, and G_Protein_ and G_Ligand_ denote the total free energies of the nine molecules bound to CYP51, respectively.

### Prediction of Pharmacological Properties

The DruLito software was used to predict the drug-like properties of the compounds. To demonstrate their pharmacological integrity, orally active drugs should have certain commonly used drug-like properties the number of rotatable bonds and Lipinski's rule violations of 5 (39) were determined during this research. By a previously described method (40), the absorption (% ABS) was calculated using the following formula:


$$\begin{array}{l} \%\text{ABS}=109-\left(0.345\times \text{TPSA}\right). \end{array}$$

### Prediction of Toxicological Properties

Since drug toxicity is an important issue, we used the admetSAR online toolbox (http://lmmd.ecust.edu.cn:8000/) to predict toxicological properties of the compounds that were critical and Predictors are helpful in drug development (41). Table 4 summarizes the data including Ames toxicity, carcinogenic properties, acute oral toxicity, acute rat toxicity, and inhibitory effects on hERGa.

### Biological Activity Predictions of the Compounds

The PASS web server (http://www.pharmaexpert.ru/passonline) was used to predict the biological activities of the designated compounds (42, 43). The PASS analysis aids in evaluating the effects of a substance only based on its molecular formula by employing multilayer atom neighbor descriptors, implying that biological behavior is solely determined by its chemical structure (44).

## Result and Discussion

### Binding Site Analysis

CASTp was used to identify the active site pockets in lanosterol-14-demethylase (CYP51). CASTp could be a web-based tool for determining the aminoalkanoic acid residues in the active site of a protein. For CYP51, the CASTp results are shown in Supplementary Figures 1, **3**. Only the amino acids within the site and their positions are given in Supplementary Table 1 for CYP51 based on the CASTp findings. According to CASTp server active amino acids are Glu173, Lys176, Tyr177, Arg179, Asp180, Ser181, Lys182, Asn183, Arg185, Asn187, Glu188, Met197, Val198, Pro201, Glu202, Ile205, Phe206, Arg218, Leu221, Asp222, Thr223, Ala226, Tyr227, Tyr229, Ser230, Leu232, Asp233, Lys234, Ile261, Tyr265, Ile309, Leu312, Met313, Gln316, His317, Ala320, Ala321, Val510, Leu512, His534, and His535 in CYP51.

### Dock Score of 1, 2, 4- Triazine and Its Derivatives Against CYP51

**Figure 2:**
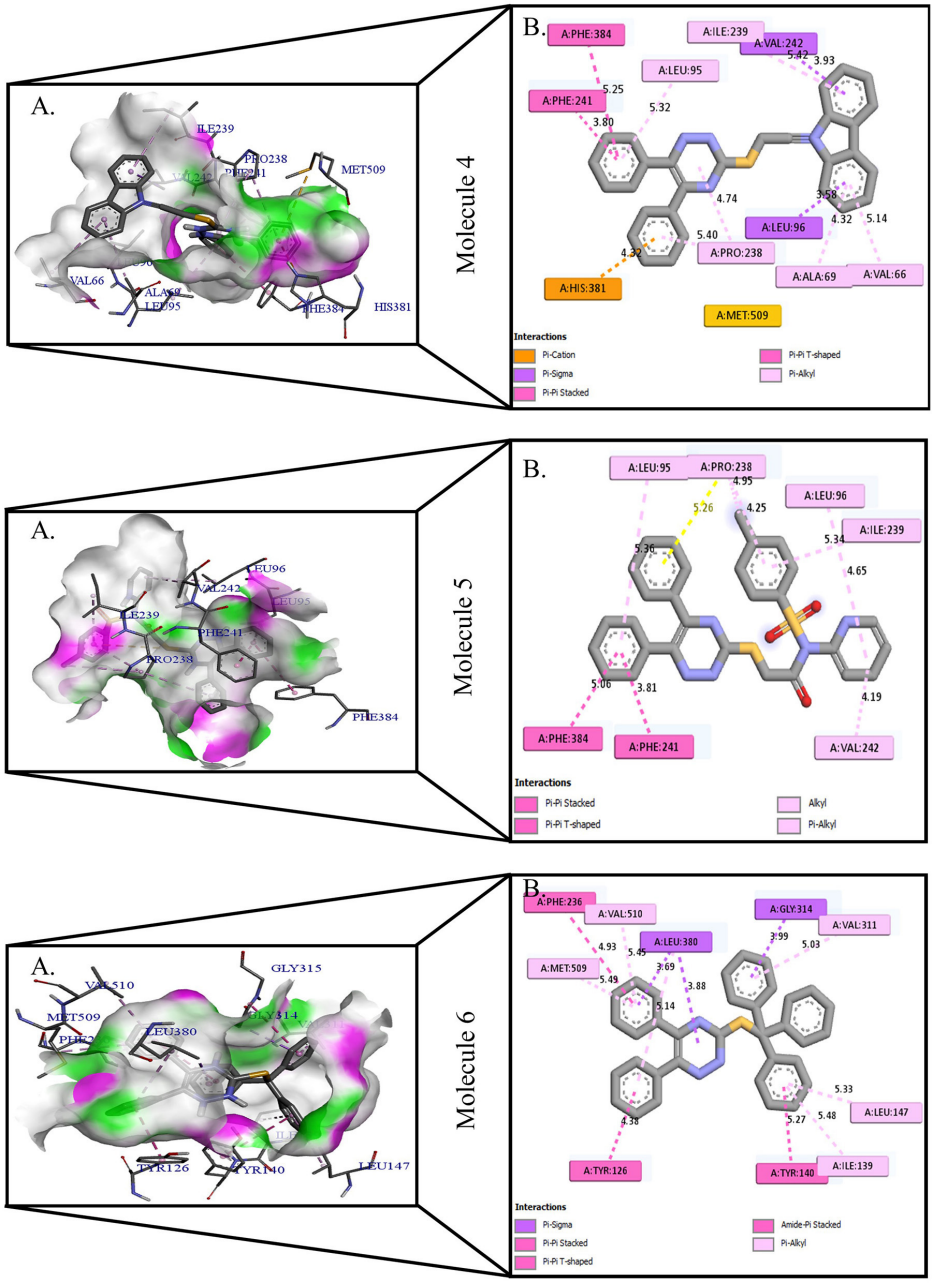
Protein-ligand presentation of CYP51 with molecules 4, 5, and 6. **(A)** 3D receptor-ligand presentation, and **(B)** 2D receptor-ligand interactions.

**Figure 3:**
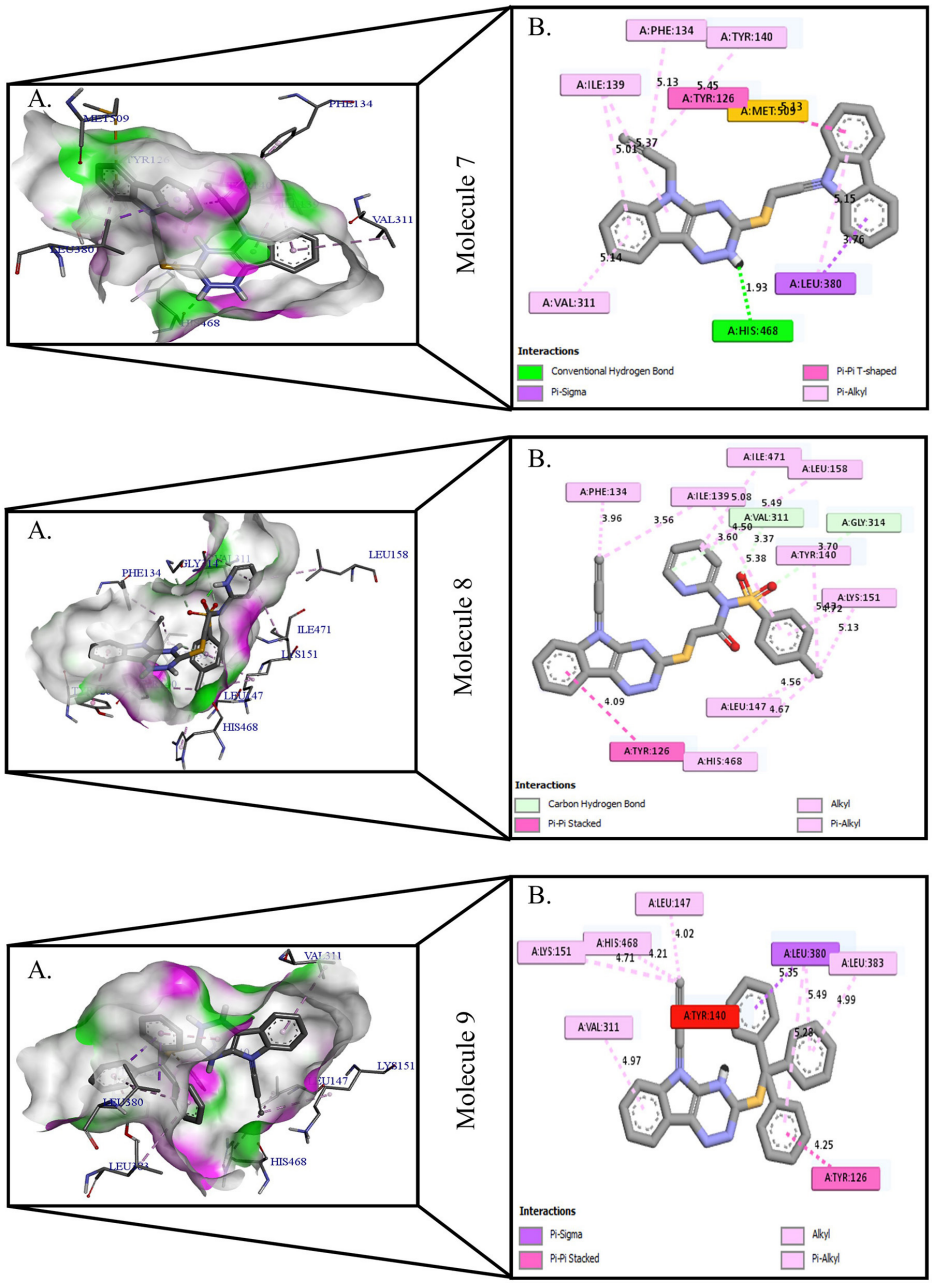
Protein-ligand presentation of CYP51 with molecules 7, 8, and 9. **(A)** 3D receptor-ligand presentation, and **(B)** 2D receptor-ligand interactions.

The crystal structure of CYP51 from *Candida albicans* (PDB id-4LXJ) was used for docking. The enhanced precision (XP) mode of lattice-based ligand docking with energetics was used to achieve the blind docking of molecules. For the docking, we used 1, 2, 4-triazine and its derivatives (molecule 1–9) (Supplementary Figure 2). Here we showed that molecules that systematically docked to lanosterol-14-demethylase (Table 1) have the separation energy (CYP51). Figures 1–3 provide a three-dimensional representation of docked complexes.

**Table 1:** Docking score (Kcal/mol) of the CYP51 with nine molecules.

**Molecules**	**Compounds names**	**ERG11** **Dock score (Kcal/mol)**
Control	Fluconazole	−8.1
1	1,2,4-Triazine	−9.5
2	1-(prop-2-en-1-yl)-1*H*-indole-2,3-dione	−8.2
3	5-(prop-2-en-1-yl)-5*H*-[1,2,4] triazino [5,6-*b*]indole-3-thiol	−8.5
4	2-((5,6-diphenyl-1,2,4-triazin-3-yl)thio)-N-(pyridin-2-yl)-N-tosylacetamide	−11.5
5	2-(2-((5,6-diphenyl-1,2,4-triazin-3-yl)thio)ethoxy)isoindoline-1,3-dione	−11.0
6	5,6-diphenyl-3-(tritylthio)-1,2,4-triazine	−11.9
7	3-((2-(9H-carbazol-9-yl)ethyl)thio)-5-allyl-5H-[1,2,4]triazino[5,6-b]indole	−10.7
8	2-((5-allyl-5*H*-[1,2,4]triazino[5,6-*b*]indol-3-yl)thio)-*N*-(pyridin-2-yl)-*N*-tosylacetamide	−10.5
9	5-allyl-3-(tritylthio)-5H-[1,2,4]triazino [5,6-b] indole	−11.5

We observed that all compounds had the best binding affinities, as indicated in Table 1, compared to the control inhibitor fluconazole (−8.1 for CYP51). For CYP51 they showed promising binding affinities with variable binding free energies in the range from −8.2 to −11.9. Based on the binding affinities, the molecules could be classified as Molecule 6 > Molecule 4 Molecule 9 > Molecule 5 > Molecule 7 > Molecule 8 > Molecule 1 > Molecule 3 > Molecule 2 for CYP51 compared to control.

### Protein-Ligand Interactions of 1, 2, 4- Triazine and Its Derivatives Against CYP51

Protein-ligand interactions are highlighted by hydrophobic interactions, hydrogen bonds, electrophoresis, and van der Waals interactions, all of which are essential for predicting ligand binding affinities with proteins. All compounds exhibited a variety of unbound and bound interactions with multiple site residues or at the edge of the site., Molecule 1 showed one conventional hydrogen interaction with the residue Thr A:507, six hydrophobic interactions (π -Alkyl and Alkyl interactions, π- π Stacked/T-Shaped Interactions) with Leu A:95, Pro A:238, Pro A: 238, Phe A:384, Phe A:241, Met A:509. Molecule 2 showed one conventional hydrogen interaction with the residue Arg A:98, six hydrophobic interactions (π -Alkyl & Alkyl interactions, π- π Stacked/T-Shaped Interactions, and Pi-Cation and Anion interactions) with His A:381, Leu A:95, Tyr A:72 Phe A:384, Phe A:241. Molecule 3 showed four conventional hydrogen interaction with the residue Thr A:507, Phe A:506, Ser A:508, Met A:509, six hydrophobic interactions (π -Alkyl and Alkyl interactions, π- π Stacked/T-Shaped Interactions, and Pi-Cation and Anion interactions) with Pro A:238, Leu A:95, Phe A:241, Phe A:384, Tyr A:72, His A:381. Molecule 4 showed ten hydrophobic interactions (π -Alkyl and Alkyl interactions, π- π Stacked/T-Shaped Interactions, and Pi-Cation and Anion interactions) with Leu A:96, Val A:242, Leu A:95, Ala A:69, Val A:66, Ile A:239, Pro A:238, Phe A:384, Phe A:241, His A:381. Molecule 5 showed seven hydrophobic interactions (π -Alkyl and Alkyl interactions, π- π Stacked/T-Shaped Interactions, and Pi-Cation and Anion interactions) with Pro A:238, Ile A:239, Val A:242, Leu A:96, Leu A:95, Phe A:384, Phe A:241. Molecule 6 showed ten hydrophobic interactions (π -Alkyl and Alkyl interactions, π- π Stacked/T-Shaped Interactions, and Pi-Cation and Anion interactions) with Gly A:314, Leu A:380, Val A:311, Val A:510, Met A:509, Leu A:147, Ile A:139, Phe A:236, Tyr A:126, Tyr A:140. Molecule 7 showed one conventional hydrogen bond with His A:468, and six hydrophobic interactions (π -Alkyl and Alkyl interactions, π- π Stacked/T-Shaped Interactions, and Pi-Cation and Anion interactions) with Leu A:380, Ile A:139, Val A:311, Tyr A:140, Phe A:134, Tyr A:126. Molecule 8 showed two carbon-hydrogen bonds with Gly A:314, Val A:311, and six hydrophobic interactions (π -Alkyl and Alkyl interactions, π- π Stacked/T-Shaped Interactions, and Pi-Cation and Anion interactions) with Phe A:134, Ile A:139, Ile A:471, Leu A:158, Tyr A:140, Tyr A:126. Molecule 9 showed eight hydrophobic interactions (π -Alkyl and Alkyl interactions, π- π Stacked/T-Shaped Interactions, and Pi-Cation and Anion interactions) with Leu A:380, Val A:311, Leu A:147, Lys A:151, His A:468, Leu A:383, Leu A:380, Tyr A:126.

All molecules showed hydrogen and hydrophobic interactions with various amino acids via the formation of conventional, carbon-hydrogen, π- stacked, or π-alkyl bonds interactions depicted in Figures 1–3 and Supplementary Table 2. It has been discovered that many amino acids are involved in hydrophobic interactions, van der Waals interactions, and hydrogen bonds. In addition, the ligands bind with the CYP51 site or the near site mainly through hydrophobic interactions. Hydrophobic interactions have effectively identified certain functional groups that are responsible for the hydrophobically producing effect of compounds with high binding affinity for target proteins and that should have a significant impact on *Candida albicans* infection.

MD simulations can be used to explain hydrogen bonds and hydrophobic properties as well as molecular processes of ligand-protein interactions depending on the flexibility of ligands or proteins. Today this method is often used in the development of biomolecules and active substances (45, 46). After molecular docking, we performed an MD simulation with targeted enzymes for all nine molecules, since all nine molecules have good dock scores compared to the standard inhibitor fluconazole.

### Molecular Dynamic Simulation

The MD simulation was performed for 10 ns on nine molecules in conjunction with lanosterol 14-demethylase (CYP51) in solute and solvent. The aim was to study the dynamic properties of lanosterol-14-demethylase (CYP51) with the nine compounds to see if structural changes related to the inhibition mechanism were observed. Conformational changes in simulated solute molecules cause density fluctuations in 10 ns. If the simulation box is always the same size, density fluctuations lead to changes in pressure. As a result, the cell is rescaled during the simulation to maintain constant cell pressure. Binding energies (Bond), binding angle energies (Angle), dihedral energies (Dihedral), planarity or false dihedral energies (planarity), van der Waals energies (VdW), electrostatic energies (Coulomb) and distribution energies are all potential energy components of all protein-ligand complexes in KJ/mol vs. time intervals. In our study, the simulation lengths of all nine molecules with complex CYP51 are between 128.34 and 128.52 (Supplementary Figure 4).

The total potential energy of CYP51 with the nine molecules was measured to determine the equilibrium and stability of the systems. Supplementary Figure 5 shows the total potential energy of CYP51 with the nine-molecule complex in 10 ns. Supplementary Figure 6 shows the surface of the dissolved CYP51 vs. the time interval of all complexes Vander Waals surface (SurfVdW), molecular surface (SurMol), and surface accessible to solvents (SurfAcc). Supplementary Figure 7 shows the surface area of the CYP51 calculated using nine molecules.

### Structural Deviations and Compactness

**Figure 4:**
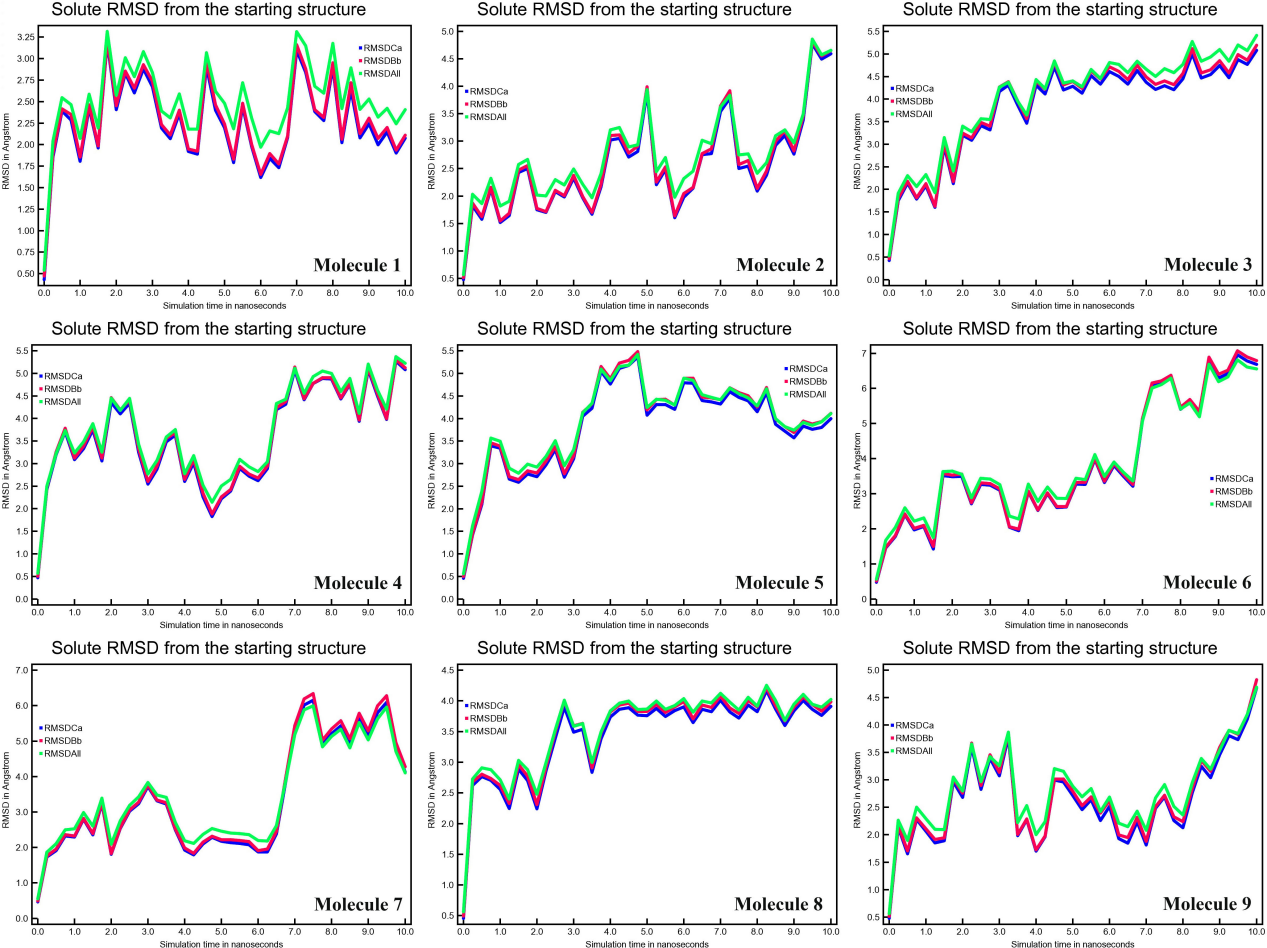
Calculations of RMSD of CYP51 and nine molecules as a function of 10 ns simulation time.

A tiny chemical can produce wide-ranging conformational changes in a protein once it has been bound. One of the most important features for analyzing protein structure changes and dynamic activity is the root mean square deviation (RMSD) (27). The complex remained stable. The calculated RMSD values of CYP51 in Å are 2.35, 5.00, 5.00, 5.00, 7.00, 6.00, 4.00, and 4.5 (Figure 4). RMSD data show significant change in the RMSD values of the CYP51 complex with nine molecules. These results show that the overall system remained stable during the MD simulation except for molecules 2–9.

**Figure 5:**
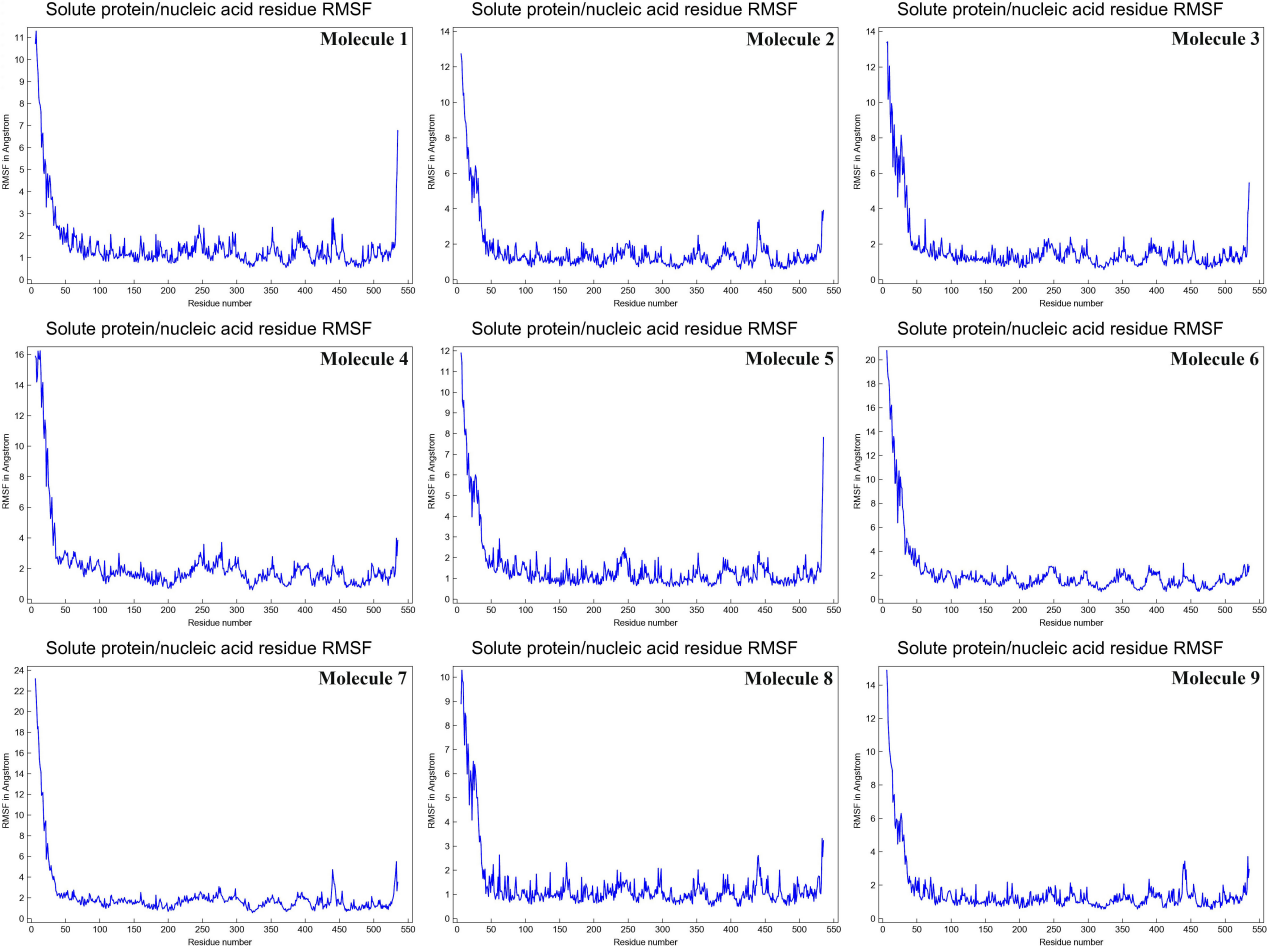
Calculations of the root mean square fluctuation per solute protein acid residue from the average RMSF of the atoms constituting the residue of CYP51 and nine molecules as a function of 10 ns simulation time.

**Figure 6:**
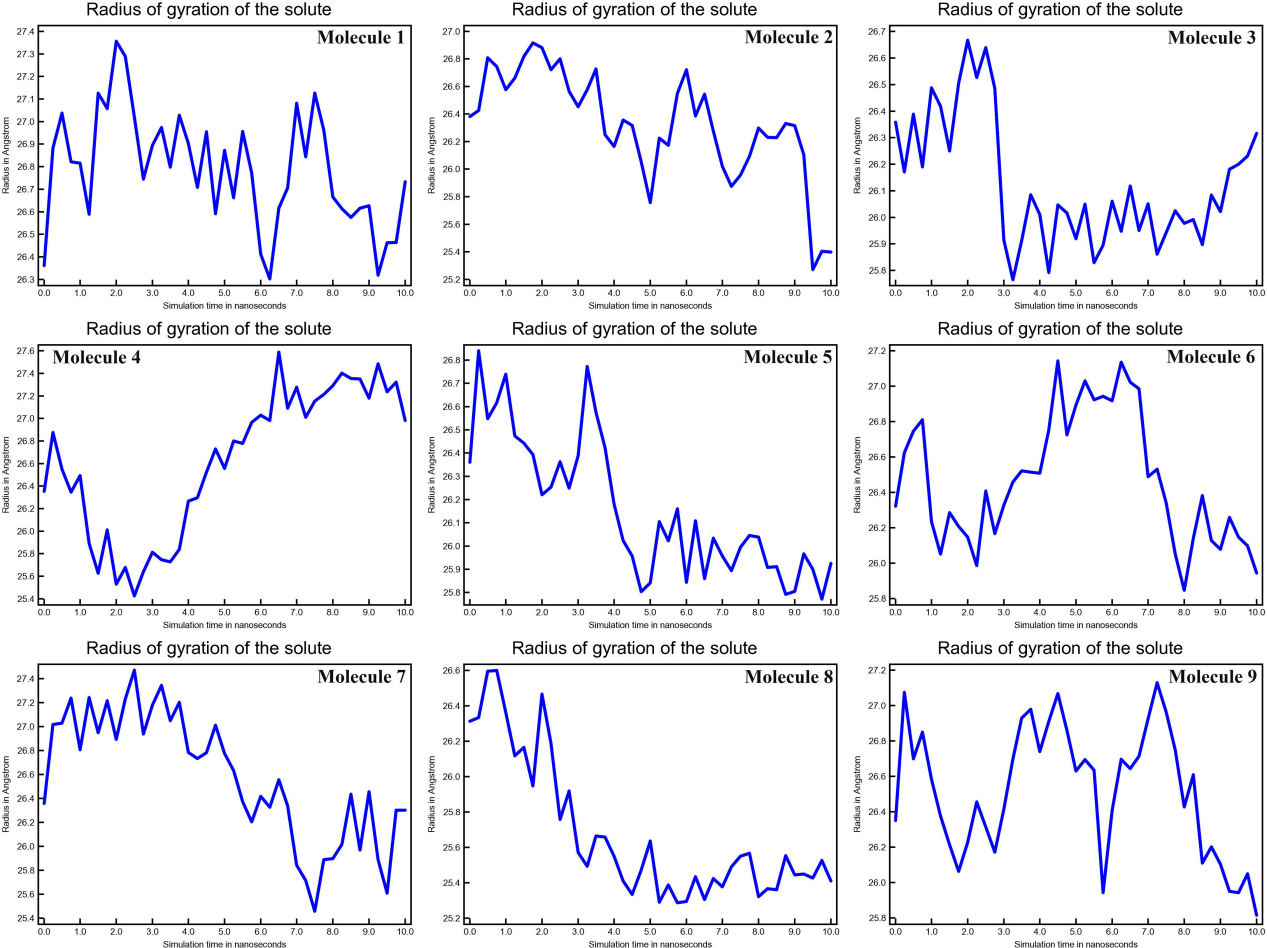
Calculations of Radius of gyration of the solute of CYP51 and nine molecules as a function of 10 ns simulation time.

The mean fluctuation of each residue was quantified as the root mean square fluctuation (RMSF) to study the residual vibrations in CYP51 before and after the binding of the nine substances (Figure 5). Random residual fluctuations were detected in CYP51 from the N-terminal to the C-terminal regions. These variations were shown in solute and solvent for each CYP51 backbone residue beyond nine. The residual fluctuations of CYP51 with nine molecular complexes were found to be 2.96, 1.93, 8.54, 2.66, 2.31, 1.44, 1.42, 2.45, and 1.26 inches. According to the RMSF map, residual fluctuations in the area where nine molecules bind show slight variations. After the nine molecules have been attached, however, CYP51 shows less increased fluctuations, which is probably due to conformational changes in the binding pocket of the protein. The radius of gyration (Rg) is a measure of the compactness and folding behavior of a protein, linked to its tertiary structure and the general state of conformation. By estimating the Rg of both CYP51 and the nine-molecule complex, we were able to determine their stability. For nine molecules the Rg stayed between 26.3–27.4, 25.2–27.0, 25.8–26.7, 25.4–27.6, 25.8–26.8, 25, 8–26.9, 25.4–27.4, 25.3–26.6, and 25.8–27.1 during the simulation period (Figure 6). No structural change was detected in CYP51 in the presence of the nine compounds, and it reached a stable Rg equilibrium, which indicates complex stability across the simulation track.

**Figure 7:**
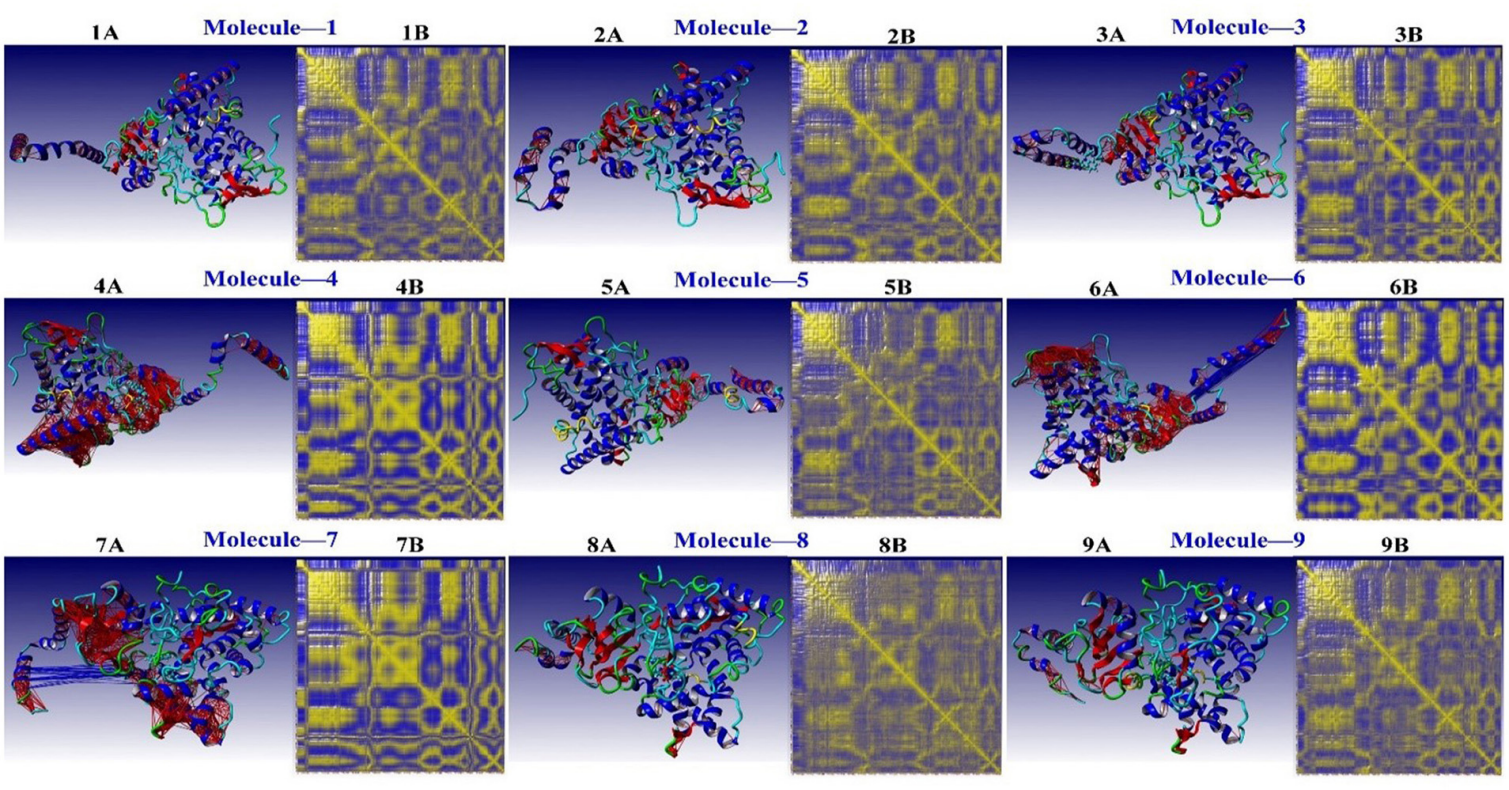
The residual correlative motion in protein structures was visualized by the dynamic cross-correlation map analysis for CYP51 [Blue and Red lines are shown between strongly, anti- and correlated pairs, and DCCM is visualized with colors ranging from blue (−1, fully anti-correlated) to yellow (+1, fully correlated)].

A complicated cross-correlation matrix represents the correlative movements of various bag remnants. Figure 7 shows the correlated residual movement of all simulated protein-ligand complexes. Heat maps with high color intensity were used to depict these linked movements between the remains of the bag. Colors ranging from blue (−1, fully anti-correlated) to yellow (+1, fully correlated) are used to visualize DCCM, with blue and red lines separating high, anti, and correlated pairings.

### Dynamics of CYP51 Interactions: Hydrogen Bond Analysis

**Figure 8:**
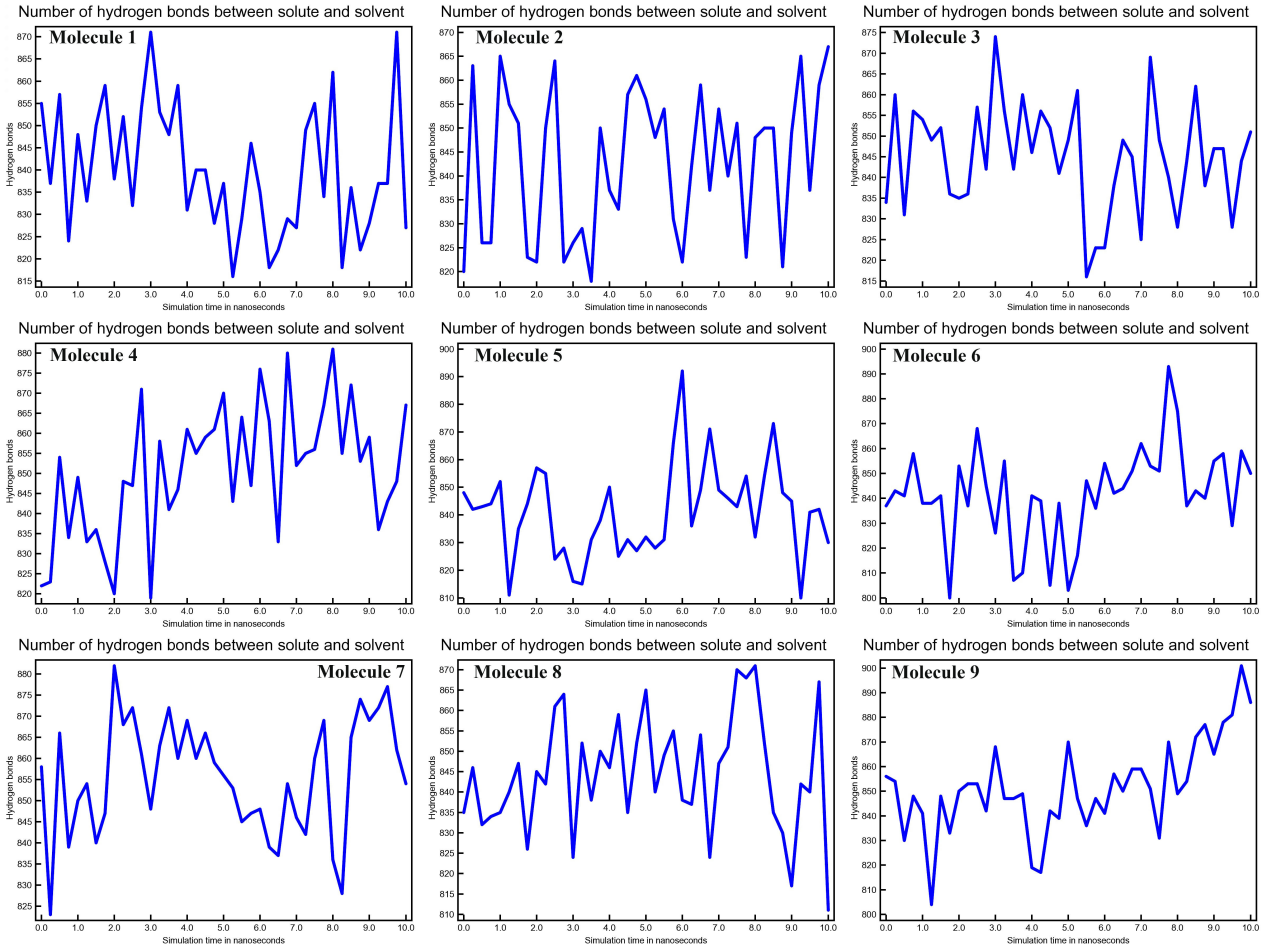
Calculations of No. of H-bonds between solute and solvent of CYP51 and nine molecules as a function of 10 ns simulation time.

The stability of the three-dimensional structure of a protein is determined by intramolecular hydrogen bonds within the molecule. By studying the stability of the protein-ligand complex, hydrogen bond analysis can be used to assess molecular recognition, directionality, and specificity of interactions (27). We calculated the kinetics of intramolecular hydrogen bond pairs within 10 ns to determine the stability of CYP51 with nine molecules docked complex, and the possibility of hydrogen bonds are 9, 6, 11, 10, 20, 8, 12, 22, and 10 of molecule 1–9, respectively The solute-solvent hydrogen bonds for all nine molecules with CYP51 complexes are shown in Figure 8 at 10 ns intervals.

### Analysis Secondary Structure Dynamics of CYP51

**Figure 9:**
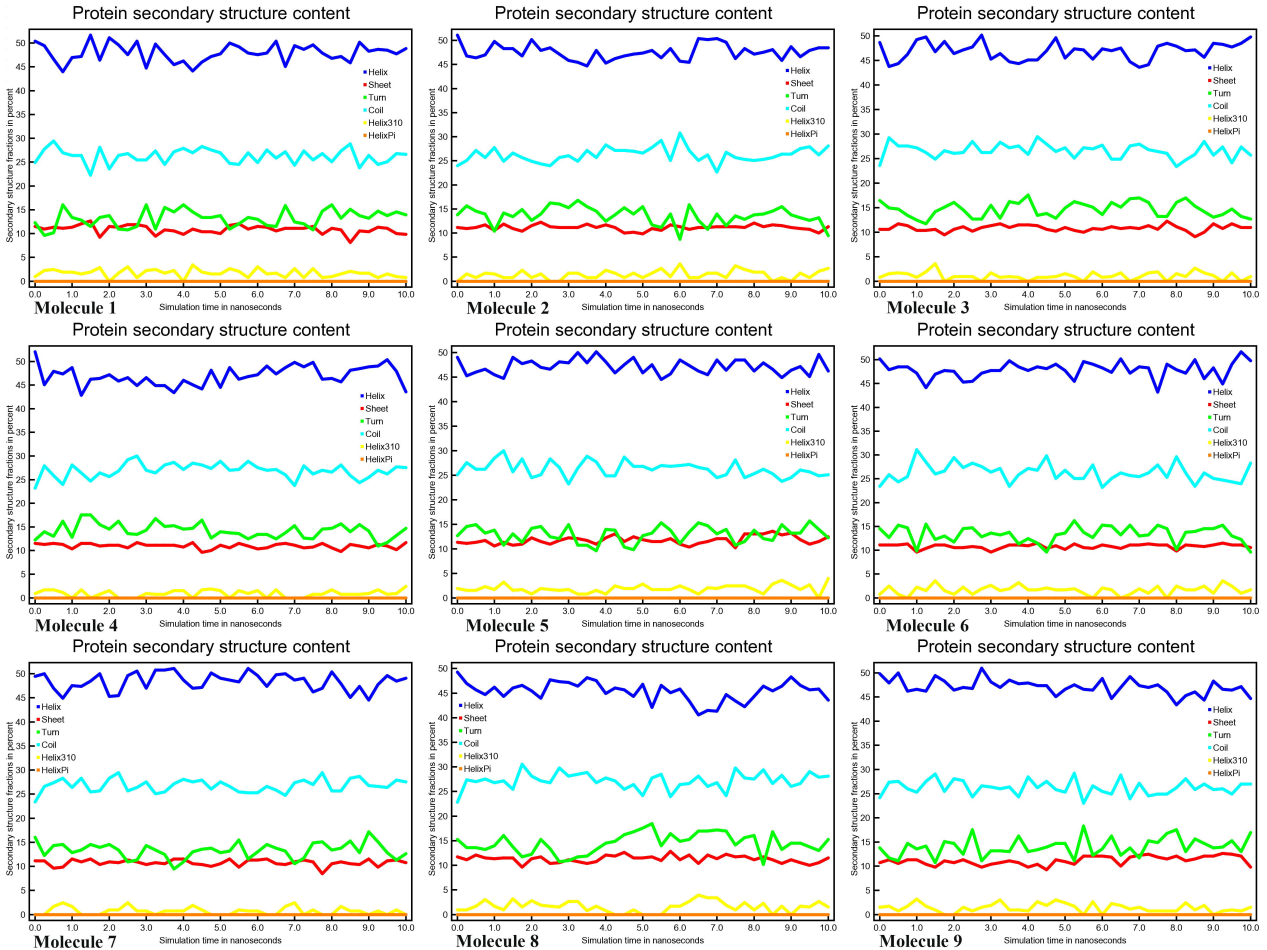
Protein secondary structure content calculations of CYP51 and nine molecules as a function of 10 ns simulation time.

**Figure 10:**
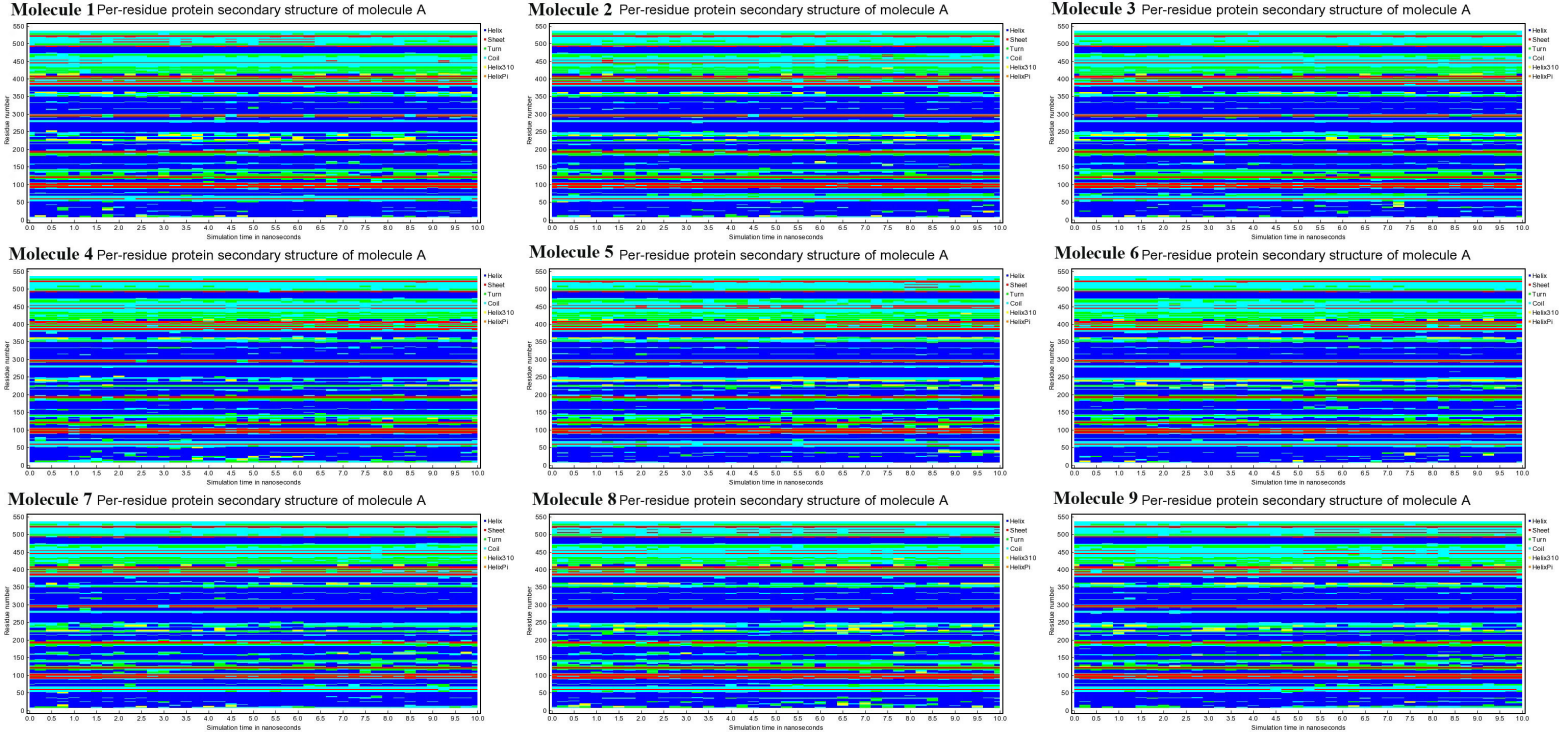
Calculations of Per-residue protein secondary structure of CYP51 and nine molecules as a function of 10 ns simulation time.

Conformational changes are caused by varying degrees of secondary structural dynamics remaining in a protein structure. Observing variations in the secondary structure composition of a polypeptide chain could help researchers better understand its conformational behavior and its folding process. We looked at the dynamics of CYP51 secondary structure content before and after all nine molecules were bound to see how stable it was. The helix, leaflet, and convolutions comprising the secondary structure of CYP51 were broken down into individual residues at each time point and the average number of residues making up the secondary structure was shown as a function of the total fraction of alpha helices, beta sheets, turns, coils, 3–10 helices, and pi helices in all nine molecular complexes ranged from 0.0 to 50% at 10 ns time intervals for all nine molecular complexes (Figure 9). Figure 10 shows the secondary structure of protein per residue for protein-ligand complexes from 00 to 550 at time intervals of 10 ns. After all nine molecules were attached, no changes in the secondary structure composition of CYP51 were found throughout 10 ns, suggesting that the complexes are very stable.

### MMPBSA Analysis

**Figure 11:**
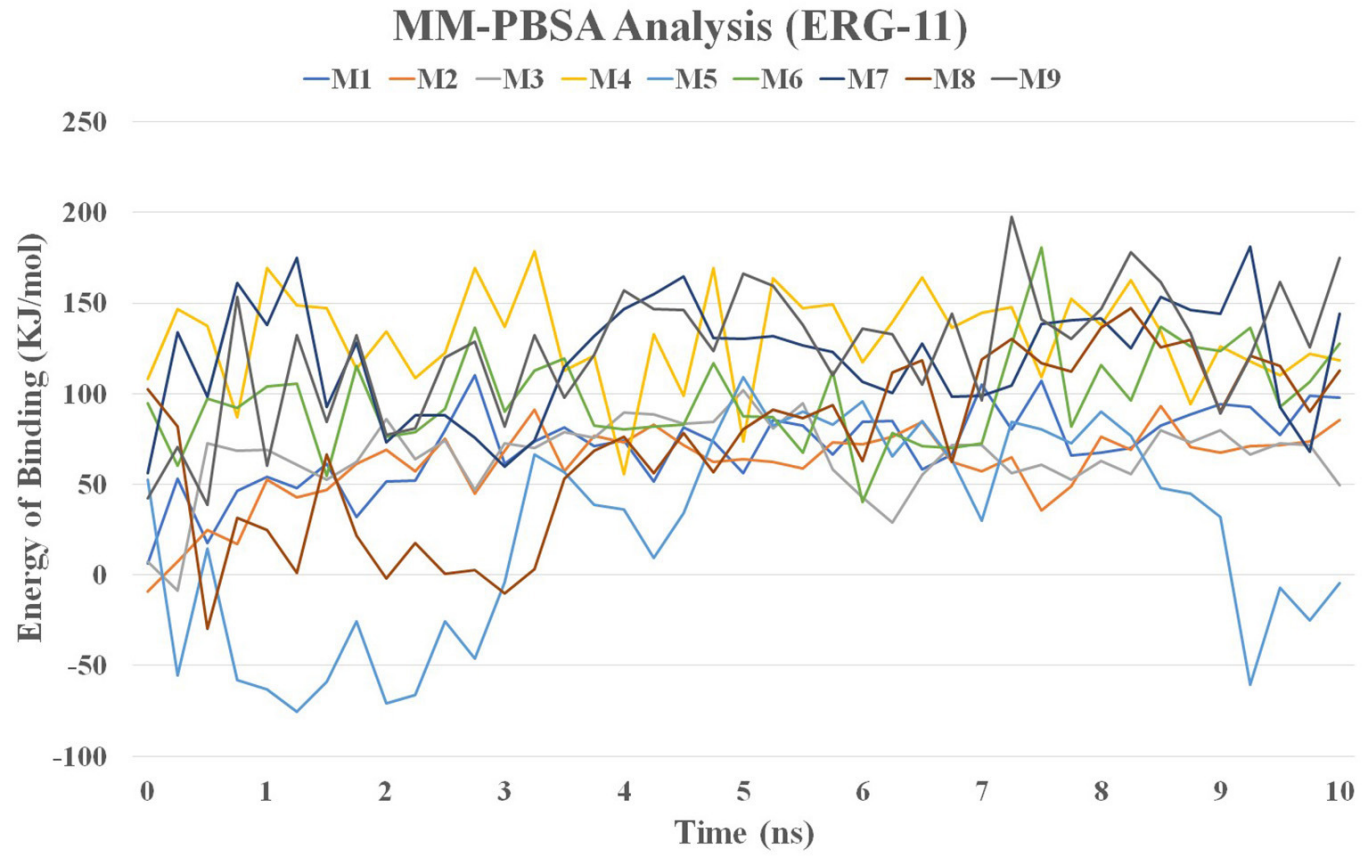
MM-PBSA analysis of nine molecules with CYP51 during 10 ns MD simulation time.

The MM / PBSA method was used to calculate the binding energy of the complex structure using ensembles derived from the MD simulation. The binding energy values for lanosterol-14-demethylase (CYP51) with nine molecules are shown in Figure 11 and Supplementary Table 3.

### Pharmacokinetics and Toxicological Properties Analysis

The ADME / T properties of ligands must be known to determine their drug similarity. The DruLito software was used to evaluate the pharmacological properties of 1, 2, 4-triazine and its derivatives (Supplementary Figure 8). It can detect molecules using drug similarity rules like Lipinski's rule, MDDR-like rule, Ghose filter, BBB similarity, CMC-50-like rule, unweighted QED, Veber filter, and weighted QED calculate and filter. Table 2 summarizes the results. Table 3 shows the expected uptake, distribution, and metabolism of 1, 2, 4-triazine and its derivatives using the admetSAR server. All compounds were found to be non-substrate for P-glycoprotein (P-gp) substrate, yes for Caco-2 permeability, yes for Blood-Brain Barrier (BBB), and non-substrate for CYP4502C9, CYP4502D6 substrate. Except molecule 2, all compounds are non-substrates for CYP4503A4. Table 3 shows that all compounds have significant CYP inhibitory promiscuity. Table 2 summarizes the results.

**Table 2:** Pharmacological properties of nine synthetic compounds according to DruLito software.

**Molecules**	**MW (g/mol)**	**logp**	**Alogp**	**HBA**	**HBD**	**TPSA (Å^**2**^)**	**%ABS**	**AMR**	**nRB**	**N Atom**	**nAcidic** **group**	**RC**	**nRigid B**	**nArom ring**	**nHB**	**SAlerts**
1.	459.98	6.418	4.734	4	0	65.62	86.36	164.53	7	36	0	6	34	6	4	2
2.	450.95	2.608	2.341	9	0	140.81	60.42	154.38	9	37	0	5	32	5	9	1
3.	413.99	5.23	5.23	5	0	68.86	85.24	143.47	6	32	0	6	31	6	5	1
4.	481.98	6.905	6.905	3	0	62.38	21.52	177.98	7	38	0	6	36	6	3	2
5.	529.94	3.095	3.095	8	0	137.57	61.53	167.84	9	39	0	5	34	5	8	1
6.	435.98	5.717	5.717	4	0	65.62	86.36	156.92	6	34	0	6	33	6	4	1
7	231.98	1.972	1.034	4	0	60.05	88.28	74.46	2	17	0	3	17	2	4	1
8	177.99	0.59	0.582	3	0	37.38	96.10	56.71	2	14	0	2	13	1	3	1
9	253.98	2.459	2.341	3	0	56.81	89.40	87.91	2	19	0	3	19	2	3	1

**Table 3:** Absorption, distribution, and metabolism of the nine molecules according to admetSAR online toolkit.

**Parameters**		**Molecules**								
		**1**	**2**	**3**	**4**	**5**	**6**	**7**	**8**	**9**
Absorption	Absorption blood-brain barrier	BBB+	BBB+	BBB+	BBB+	BBB+	BBB+	BBB+	BBB+	BBB+
	Human intestinal absorption	HIA+	HIA+	HIA+	HIA+	HIA+	HIA+	HIA+	HIA+	HIA+
	Caco-2 permeability	Caco2+	Caco2+	Caco2+	Caco2+	Caco2+	Caco2+	Caco2+	Caco2+	Caco2+
	P-glycoprotein substrate	Non-Substrate	Non-Substrate	Non-Substrate	Non-Substrate	Non-Substrate	Non-Substrate	Non-Substrate	Non-Substrate	Non-Substrate
	P-glycoprotein inhibitor	Non-Inhibitor	Non-Inhibitor	Non-Inhibitor	Inhibitor	Non-Inhibitor	Non-Inhibitor	Non-Inhibitor	Non-Inhibitor	Non-Inhibitor
	Renal organic cation transporter	Non-Inhibitor	Non-Inhibitor	Non-Inhibitor	Inhibitor	Non-Inhibitor	Non-Inhibitor	Non-Inhibitor	Non-Inhibitor	Non-Inhibitor
Distribution	Subcellular localization	Mitochondria	Mitochondria	Mitochondria	Mitochondria	Mitochondria	Mitochondria	Mitochondria	Plasma membrane	Mitochondria
Metabolism	CYP450 2C9 substrate	Non-substrate	Non-substrate	Non-substrate	Non-substrate	Non-substrate	Non-substrate	Non-substrate	Non-substrate	Non-substrate
	CYP450 2D6 substrate	Non-substrate	Non-substrate	Non-substrate	Non-substrate	Non-substrate	Non-substrate	Non-substrate	Non-substrate	Non-substrate
	CYP450 3A4 substrate	Non-substrate	Substrate	Non-substrate	Non-substrate	Non-substrate	Non-substrate	Non-substrate	Non-substrate	Non-substrate
	CYP450 1A2 inhibitor	Inhibitor	Inhibitor	Inhibitor	Inhibitor	Non-inhibitor	Inhibitor	Inhibitor	Non-inhibitor	Inhibitor
	CYP450 2C9 inhibitor	Inhibitor	Non-inhibitor	Non-inhibitor	Non-inhibitor	Inhibitor	Inhibitor	Non-inhibitor	Inhibitor	Non-inhibitor
	CYP450 2D6 inhibitor	Non-inhibitor	Non-inhibitor	Non-inhibitor	Non-inhibitor	Non-inhibitor	Non-inhibitor	Non-inhibitor	Non-inhibitor	Non-inhibitor
	CYP450 2C19 inhibitor	Inhibitor	Non-inhibitor	Inhibitor	Inhibitor	Inhibitor	Inhibitor	Inhibitor	Inhibitor	Inhibitor
	CYP450 3A4 inhibitor	Inhibitor	Non-inhibitor	Inhibitor	Inhibitor	Non-inhibitor	Non-inhibitor	Non-inhibitor	Inhibitor	Inhibitor
	CYP inhibitory promiscuity	High CYP inhibitory promiscuity	High CYP inhibitory promiscuity	High CYP inhibitory promiscuity	High CYP inhibitory promiscuity	High CYP inhibitory promiscuity	High CYP inhibitory promiscuity	High CYP inhibitory promiscuity	High CYP inhibitory promiscuity	High CYP inhibitory promiscuity

SWISS ADME boiled egg diagram (Supplementary Figure 9) enables the evaluation of HIA as a function of the position of the ten molecules within the WLOGP-vs.-TPSA reference. Molecule 1 (control), molecule 8 are predicted to be absorbed by the gastrointestinal tract (white region) but not penetrate the brain (egg yolk). Molecules 1–10 are not subject to any active efflux (redpoint). The admet website was used to predict the toxicological properties of all compounds (**Table 4**). The results indicated that, except for molecule 3, none of the compounds posed an AMES toxicity problem and that all of the molecules are non-carcinogenic. On the other hand, all compounds were found to be weak inhibitors of the human ether-a-go-go-related gene (HERGa) and have a low risk of acute toxicity. In accordance with the expected acute oral toxicity, all compounds are classified in Class III based on their expected acute oral toxicity (Table 4). It is possible that the combination of these chemicals can be predicted, and it is a good idea to stop mycosis.

**Table 4:** Toxicological properties of the molecules according to admetSAR online toolkit.

**Parameters**	**Molecules**								
	**1**	**2**	**3**	**4**	**5**	**6**	**7**	**8**	**9**
Ames toxicity	Non-AMES toxic	Non-AMES toxic	Non-AMES toxic	Non-AMES toxic	Non-AMES toxic	Non-AMES toxic	Non-AMES toxic	Non-AMES toxic	Non-AMES toxic
Carcinogens	Non-carcinogens	Non-carcinogens	Non-carcinogens	Non-carcinogens	Non-carcinogens	Non-carcinogens	Non-carcinogens	Non-carcinogens	Non-carcinogens
Acute oral toxicity	III	III	III	II	III	III	III	III	III
Rat acute toxicity (LD50, mol/kg)	2.0935	2.3903	2.2992	2.2665	2.2857	2.2029	2.2397	2.5164	2.3316
hERG^a^	Weak Inhibitor/non-inhibitor	Weak Inhibitor/non-inhibitor	Weak Inhibitor/non-inhibitor	Weak Inhibitor/non-inhibitor	Weak Inhibitor/non-inhibitor	Weak Inhibitor/non-inhibitor	Weak Inhibitor/non-inhibitor	Weak Inhibitor/non-inhibitor	Weak Inhibitor/non-inhibitor
Carcinogenicity (three-class)	Non-required	Non-required	Non-required	Non-required	Non-required	Non-required	Non-required	Non-required	Non-required
Caco-2 permeability (LogPapp, cm/s)	1.8374	1.6378	1.1742	1.3147	0.9497	1.9877	1.2075	0.7581	1.3479

a *Human ether-a-go-go-related gene*.

Antifungal and antibacterial activities against strains of 1, 2, 4-triazine and its derivatives were carried out by Majid et al. (21), and these compounds showed good antifungal activity compared to control fluconazole by *in vitro* activity assays.

Computational engineering can filter the best ligands from a wide variety of compounds, simplifying the drug development process. After that, further clinical research with experimental animals and further *in vivo* studies are required. All of the basic information needed to develop novel antifungal treatments to block Ergosterol biosynthesis will be included in the study. Diseases caused by eukaryotic organisms such as fungi are more difficult to treat than infections caused by bacteria. Antifungal drugs that can discover unique targets that are not shared with human hosts are few and far between. Due to the lack of chitin structure in human cells, the fungal cell wall remains an untapped therapeutic target for selective antimycotics (47, 48). Most treatments are aimed at treating fungal infections and target the Ergosterol production route or its end product Ergosterol, a membrane sterol found only in fungi. It is the primary sterol and is therefore needed for fungal cell development and proper membrane function. It contributes to the correct activity of membrane-bound enzymes and also acts as a bioregulator of membrane fluidity, asymmetry, and integrity (49). As a result, efficient antifungal drugs and the discovery of a potential target to treat them will become possible. Diseases are urgently needed. One of the most important proteins in mycoses is lanosterol-14-demethylase (CYP51). As a result of this discovery, this protein has been identified as a potential therapeutic target.

### Biological Activity Predictions of Compounds

The PASS webserver was used to confirm the biological activity prediction, resulting in the selected compounds having the same biological activities. This study demonstrated that the molecules series 1–9 have antifungal therapeutic properties, validating prior findings except molecules 4–9. Several clinical trials assessing the use of anti-inflammatory drugs in combination with chemotherapeutic drugs for cancer prevention and treatment have been conducted, with antifungal therapies showing promising efficacy and toxicity findings. Preclinical and clinical research to assess the antifungal properties and mode of action of new medicines are underway. With Pa ranging from 0.296 to 0.240, the molecules demonstrated strong antifungal inhibitory potential predictions when Pa > Pi. Supplementary Table 4 shows the projected activity of the antifungal medications with the highest Pa values.

## Conclusion

This study has utilized comprehensive *in-silico* techniques for determining the anti-fungal activity of derivatives of 1, 2, 4-triazine with CYP51. Compared to the conventional inhibitor fluconazole, all derivatives of 1, 2, 4-triazine showed stronger binding affinities to the target protein. We discovered that all compounds can act as inhibitors against a specific protein of *Candida albicans*, based on molecular docking and chemical bonding, hydrophobic interactions, and van der Waals interactions nine molecules with the protein Lanosterol 14-demethylase (CYP51). The RMSD and RMSF values obtained by molecular dynamic simulation have proved that the selection of docked complexes seems to be correct. The amino acid residues associated with the binding pose of the ligand with the protein change over the simulation time showing the point mutation in the protein. It also conveys the availability of polar and non-polar interactions. Their drug-like properties have been demonstrated through physicochemical, pharmacokinetic, and toxicological characteristics, which have proven them to be safe sources of drugs. Overall, we conclude that 1, 2, 4-triazine and its derivatives, either alone or in combination with the different compounds, can target CYP51 or may be beneficial for future antifungal drug development.

## Data Availability Statement

The original contributions presented in the study are included in the article/supplementary materials, further inquiries can be directed to the corresponding author/s.

## Author Contributions

AV and AM designed and conceptualized the research. MH and SA performed MD simulation analysis. AV, AM, MA, and AB interpreted the data. AV, SU, NV, and MdA prepared, reviewed, and edited the original draft. All authors contributed to the article and approved the submitted version.

## Conflict of Interest

The authors declare that the research was conducted in the absence of any commercial or financial relationships that could be construed as a potential conflict of interest. The handling editor DJ declared a past collaboration with the author AB.

## Publisher's Note

All claims expressed in this article are solely those of the authors and do not necessarily represent those of their affiliated organizations, or those of the publisher, the editors and the reviewers. Any product that may be evaluated in this article, or claim that may be made by its manufacturer, is not guaranteed or endorsed by the publisher.

## References

[1] Tang H Zheng C Lv J Wu J Li Y Yang H. Synthesis and antifungal activities in vitro of novel pyrazino [2, 1-a] isoquinolin derivatives. Bioorganic Med Chem Lett. (2010) 20:979–82. 10.1016/j.bmcl.2009.12.050 20036534

[2] Wang W Sheng C Che X Ji H Miao Z Yao J. Design, synthesis, and antifungal activity of novel conformationally restricted triazole derivatives. Arch Pharm. (2009) 342:732–9. 10.1002/ardp.200900103 19899102

[3] Ghabbour HA Qabeel MM Eldehna WM Al-Dhfyan A Abdel-Aziz HA. Design, synthesis, and molecular docking of 1-(1-(4-chlorophenyl)-2-(phenylsulfonyl) ethylidene)-2-phenylhydrazine as potent nonazole anticandidal agent. J. Chem. (2014) 2014:154357. 10.1155/2014/154357

[4] Emami S Banipoulad T Irannejad H Foroumadi A Falahati M Ashrafi-Khozani M. Imidazolylchromanones containing alkyl side chain as lanosterol 14α-demethylase inhibitors: synthesis, antifungal activity and docking study. J Enzyme Inhib Med Chem. (2014) 29:263–71. 10.3109/14756366.2013.776554 23488742

[5] Kankate RS Gide PS Belsare DP. Design, synthesis and antifungal evaluation of novel benzimidazole tertiary amine type of fluconazole analogues. Arab J Chem. (2019) 12:2224–35. 10.1016/j.arabjc.2015.02.002

[6] Ahmad A Khan A Manzoor N Khan LA. Evolution of ergosterol biosynthesis inhibitors as fungicidal against Candida. Microb Pathog. (2010) 48:35–41. 10.1016/j.micpath.2009.10.001 19835945

[7] Sheng C Miao Z Ji H Yao J Wang W Che X. Three-dimensional model of lanosterol 14α-demethylase from *Cryptococcus neoformans*: active-site characterization and insights into azole binding. Antimicrob Agents Chemother. (2009) 53:3487–95. 10.1128/AAC.01630-08 19470512 PMC2715644

[8] Zhang Q Li D Wei P Zhang J Wan J Ren Y. Structure-based rational screening of novel hit compounds with structural diversity for cytochrome P450 sterol 14α-demethylase from *Penicillium digitatum*. J Chem Inf Model. (2010) 50:317–25. 10.1021/ci900425t 20088581

[9] Jacob KS Ganguly S Kumar P Poddar R Kumar A. Homology model, molecular dynamics simulation and novel pyrazole analogs design of Candida albicans CYP450 lanosterol 14 α-demethylase, a target enzyme for antifungal therapy. J Biomol Struct Dyn. (2017) 35:1446–63. 10.1080/07391102.2016.1185380 27142238

[10] Dogan IS Saraç S Sari S Kart D Gökhan SE Vural I. New azole derivatives showing antimicrobial effects and their mechanism of antifungal activity by molecular modeling studies. Eur J Med Chem. (2017) 130:124–138. 10.1016/j.ejmech.2017.02.035 28242548

[11] Reddy KK Singh SK Tripathi SK Selvaraj C Suryanarayanan V. Shape and pharmacophore-based virtual screening to identify potential cytochrome P450 sterol 14α-demethylase inhibitors. J Recept Signal Transduct Res. (2013) 33:234–43. 10.3109/10799893.2013.789912 23638723

[12] Stana A Vodnar DC Tamaian R Pîrnău A Vlase L Ionuţ I. Design, synthesis and antifungal activity evaluation of new thiazolin-4-ones as potential lanosterol 14α-demethylase inhibitors. Int J Mol Sci. (2017) 18:177. 10.3390/ijms18010177 28106743 PMC5297809

[13] Warrilow AG Mullins JG Hull CM Parker JE Lamb DC Kelly DE. S279 point mutations in Candida albicans sterol 14-α demethylase (CYP51) reduce in vitro inhibition by fluconazole. Antimicrob Agents Chemother. (2012) 56:2099–107. 10.1128/AAC.05389-11 22252802 PMC3318376

[14] Apeh VO Njoku OU Nwodo FOC Chukwuma IF Emmanuel AA. In silico drug-like properties prediction and in vivo antifungal potentials of *Citrullus lanatus* seed oil against *Candida albicans*. Arab J Chem. (2022) 15:103578. 10.1016/j.arabjc.2021.103578

[15] Sagatova AA Keniya MV Wilson RK Sabherwal M Tyndall JD Monk BC. Triazole resistance mediated by mutations of a conserved active site tyrosine in fungal lanosterol 14α-demethylase. Sci Rep. (2016) 6:1–11. 10.1038/srep26213 27188873 PMC4870556

[16] Sheng C Zhang W Zhang M Song Y Ji H Zhu J. Homology modeling of lanosterol 14α-demethylase of *Candida albicans* and *Aspergillus fumigatus* and insights into the enzyme-substrate interactions. J Biomol Struct Dyn. (2004) 22:91–9. 10.1080/07391102.2004.10506984 15214809

[17] Jiang Y Zhang J Cao Y Chai X Zou Y Wu Q. Synthesis, *in vitro* evaluation and molecular docking studies of new triazole derivatives as antifungal agents. Bioorganic Med Chem Lett. (2011) 21:4471–5. 10.1016/j.bmcl.2011.06.008 21737273

[18] Sheehan DJ Hitchcock CA Sibley CM. Current and emerging azole antifungal agents. Clin Microbiol Rev. (1999) 12:40–79. 10.1128/CMR.12.1.40 9880474 PMC88906

[19] Jadhav AK Khan PK Karuppayil SM. Phytochemicals as potential inhibitors of lanosterol 14 A-demethylase (Cyp51) enzyme: an *in silico* study on sixty molecules. Int J Appl Pharm. (2020) 12:18–30. 10.22159/ijap.2020.v12s4.40100

[20] Verma AK Maurya SK Kumar A Barik M Yadav V Umar B. Inhibition of multidrug resistance property of *Candida albicans* by natural compounds of *Parthenium hysterophorus* L. An *in-silico* approach. J Pharmacogn Phytochem. (2020) 9:55–64. 10.22271/phyto.2020.v9.i3a.11480

[21] Majid A Ashid M Hussain N. a convenient synthesis and reactions of some substituted 1, 2, 4-triazine, and their derivatives with carbazole, sulfonamide and trityl chloride moiety of biological interest. EJMCM. (2020) 7:994–1004.

[22] RCSB Protein Data Bank. Available online at: https://www.rcsb.org/ (accessed December 05, 2021).

[23] Yuan S Chan HS Hu Z. Using PyMOL as a platform for computational drug design. Wiley Interdiscip Rev Comput Mol Sci. (2017) 7:e1298. 10.1002/wcms.129821053052

[24] RCSB PubChem Database. Available online at: https://pubchem.ncbi.nlm.nih.gov/ (accessed December 05, 2021).

[25] Tian W Chen C Lei X Zhao J Liang J. CASTp 3.0: computed atlas of surface topography of proteins. Nucleic Acids Res. (2018) 46:W363–7. 10.1093/nar/gky473 29860391 PMC6031066

[26] Trott O Olson AJ. AutoDock Vina: improving the speed and accuracy of docking with a new scoring function, efficient optimization, and multithreading. J Comput Chem. (2010) 31:455–61. 10.1002/jcc.21334 19499576 PMC3041641

[27] Verma AK Ahmed SF Hossain MS Bhojiya AA Mathur A Upadhyay SK. Molecular docking and simulation studies of flavonoid compounds against PBP-2a of methicillin-resistant *Staphylococcus aureus*. J Biomol Struct Dyn. (2021) 1-17. 10.1080/07391102.2021.1944911 34243699

[28] Lawal M Verma AK Umar IA Gadanya AM Umar B Yahaya N. Analysis of new potent anti-diabetic molecules from phytochemicals of pistia strateotes with Sglt1 and G6pc proteins of homo sapiens for treatment of diabetes mellitus. In Silico Approach IOSR JPBS. (2020) 15:59–73. 10.9790/3008-1504025973

[29] Maurya SK Maurya AK Mishra N Siddique HR. Virtual screening, ADME/T, and binding free energy analysis of anti-viral, anti-protease, and anti-infectious compounds against NSP10/NSP16 methyltransferase and main protease of SARS CoV-2. J Recept Signal Transduct Res. (2020) 40:605–12. 10.1080/10799893.2020.1772298 32476594 PMC7284148

[30] Biovia DS. Discovery Studio Modeling Environment (2017) San Diego: DassaultSystémes.

[31] Krieger E Vriend G. New ways to boost molecular dynamics simulations. J Comput Chem. (2015) 36:996–1007. 10.1002/jcc.23899 25824339 PMC6680170

[32] Ahmad S Raza S Uddin R Azam SS. Binding mode analysis, dynamic simulation and binding free energy calculations of the MurF ligase from *Acinetobacter baumannii*. J Mol Graph Model. (2017) 77:72–85. 10.1016/j.jmgm.2017.07.024 28843462

[33] Kumar SP Patel CN Jha PC Pandya HA. Molecular dynamics-assisted pharmacophore modeling of caspase-3-isatin sulfonamide complex: recognizing essential intermolecular contacts and features of sulfonamide inhibitor class for caspase-3 binding. Comput Biol Chem. (2017) 71:117–28. 10.1016/j.compbiolchem.2017.08.006 29153890

[34] Tietze D Kaufmann D Tietze AA Voll A Reher R König G Hausch F. Structural and dynamical basis of G protein inhibition by YM-254890 and FR900359: an inhibitor in action. J Chem Inf Model. (2019) 59:4361–73. 10.1021/acs.jcim.9b00433 31539242

[35] Elfiky AA Azzam EB. Novel guanosine derivatives against MERS CoV polymerase: an *in silico* perspective. J Biomol Struct Dyn. (2020) 39:2923–31. 10.1080/07391102.2020.1758789 32306854 PMC7189410

[36] Enayatkhani M Hasaniazad M Faezi S Guklani H Davoodian P Ahmadi N. Reverse vaccinology approach to design a novel multi-epitope vaccine candidate against COVID-19: an *in silico* study. J Biomol Struct Dyn. (2020) 39:2857–72. 10.1080/07391102.2020.1756411 32295479 PMC7196925

[37] Pant S Singh M Ravichandiran V Murty USN Srivastava HK. Peptide-like and small-molecule inhibitors against Covid-19. J Biomol Struct Dyn. (2020) 39:2904–13. 10.1080/07391102.2020.1757510 32306822 PMC7212534

[38] Homeyer N Gohlke H. Free energy calculations by the molecular mechanics Poisson– Boltzmann surface area method. Mol Inform. (2012) 31:114–22. 10.1002/minf.201100135 27476956

[39] Lipinski CA Lombardo F Dominy BW Feeney PJ. Experimental and computational approaches to estimate solubility and permeability in drug discovery and development settings. Adv Drug Deliv Rev. (1997) 46:3–26. 10.1016/S0169-409X(96)00423-1 11259830

[40] Zhao YH Abraham MH Le J Hersey A Luscombe CN Beck G. Rate-limited steps of human oral absorption and QSAR studies. Pharm Res. (2002) 19:1446–57. 10.1023/A:1020444330011 12425461

[41] Cheng F Li W Zhou Y Shen J Wu Z Liu G. admetSAR: a comprehensive source and free tool for assessment of chemical ADMET properties. J Chem Inf Model. (2012) 52:3099–105. 23092397 10.1021/ci300367a

[42] Ali S Khan FI Mohammad T Lan D Hassan M Wang Y. Identification and evaluation of inhibitors of lipase from Malassezia restricta using virtual high-throughput screening and molecular dynamics studies. Int J Mol Sci. (2019) 20:884. 10.3390/ijms20040884 30781686 PMC6412828

[43] Gulzar M Syed SB Khan FI Khan P Ali S Hasan GM. Elucidation of interaction mechanism of ellagic acid to the integrin linked kinase. Int J Biol Macromol. (2019) 122:1297–304. 10.1016/j.ijbiomac.2018.09.089 30227205

[44] Ramos RS Macêdo WJ Costa JS da Silva CHDP Rosa JM da Cruz JN. Potential inhibitors of the enzyme acetylcholinesterase and juvenile hormone with insecticidal activity: study of the binding mode via docking and molecular dynamics simulations. J Biomol Struct Dyn. (2020) 38:4687–709. 10.1080/07391102.2019.1688192 31674282

[45] Barril X Luque FJ. Molecular simulation methods in drug discovery: a prospective outlook. J Comput Aided Mol Des. (2012) 26:81–6. 10.1007/s10822-011-9506-1 22160626

[46] Tatar G Ozyurt E Turhan K. Computational drug repurposing study of the RNA binding domain of SARS-CoV-2 nucleocapsid protein with antiviral agents. Biotechnol Prog. (2021) 37:e3110. 10.1002/btpr.3110 33314794 PMC7883068

[47] Malik MA Al-Thabaiti SA Malik MA. Synthesis, structure optimization and antifungal screening of novel tetrazole ring bearing acyl-hydrazones. Int J Mol Sci. (2012) 13:10880–98. 10.3390/ijms130910880 23109826 PMC3472718

[48] Dhingra S Cramer RA. Regulation of sterol biosynthesis in the human fungal pathogen *Aspergillus fumigatu*s: opportunities for therapeutic development. Front Microbiol. (2017) 8:92. 10.3389/fmicb.2017.00092 28203225 PMC5285346

[49] Lupetti A Danesi R Campa M Del Tacca M Kelly S. Molecular basis of resistance to azole antifungals. Trends Mol Med. (2002) 8:76–81. 10.1016/s1471-4914(02)02280-3 11815273

