# Supplementary material for: Identification of 1, 2, 4-Triazine and Its Derivatives Against Lanosterol 14-Demethylase (CYP51) Property of Candida albicans: Influence on the Development of New Antifungal Therapeutic Strategies
Source: Front Med Technol. 2022 Mar 28;4:845322. doi: 10.3389/fmedt.2022.845322 (PMC8996309; doi:10.3389/fmedt.2022.845322)
Supplement: Supplementary file 1 [file Data_Sheet_1.docx]

**Supplementary Information**


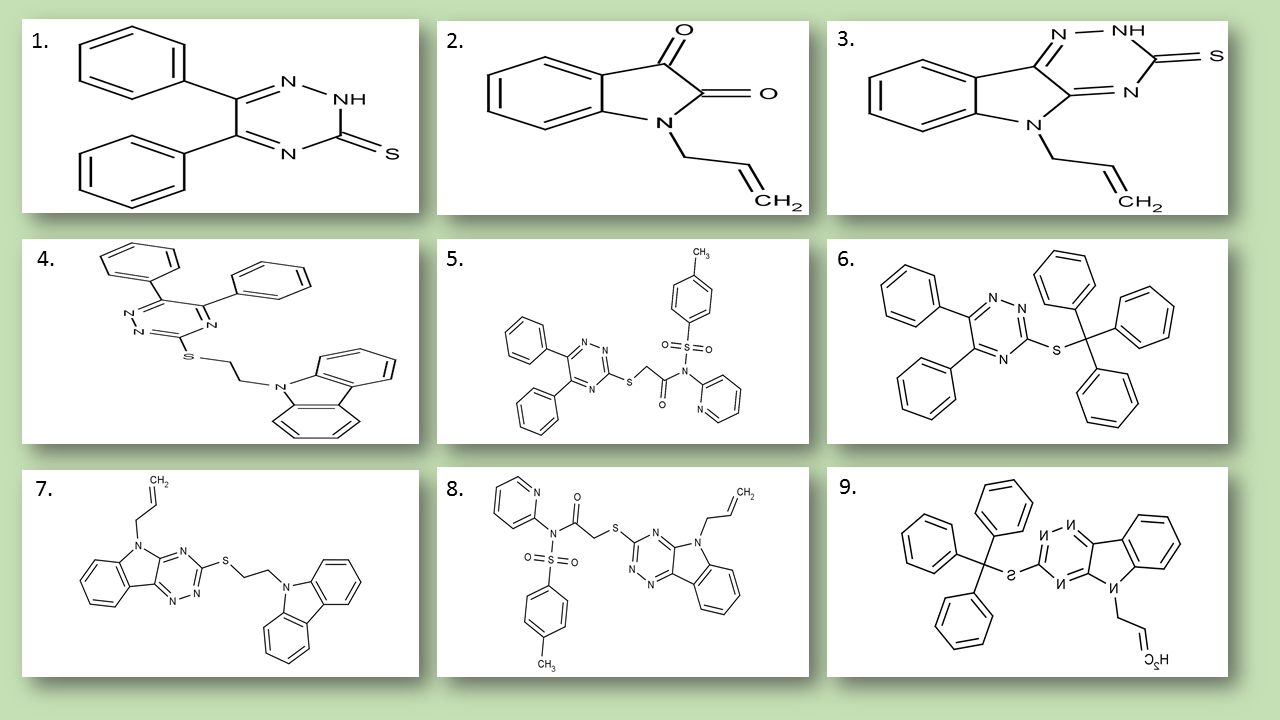


**Figure S1.** Chemical structures of 1, 2, 4-Triazine and their derivatives.


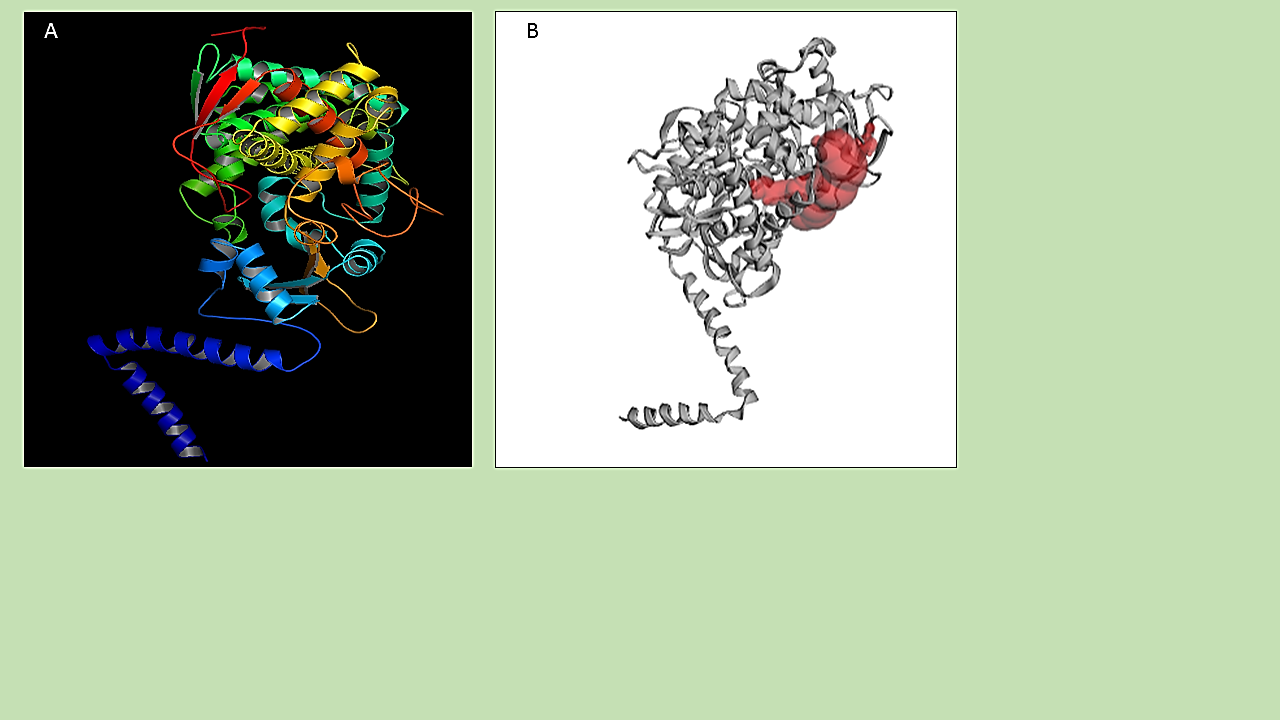


**Figure S2.** The Structure of Lanosterol 14 - demethylase (CYP51) (PDB id- 4LXJ) (A. Ribbon Presentation; B. Active site of ERG11).


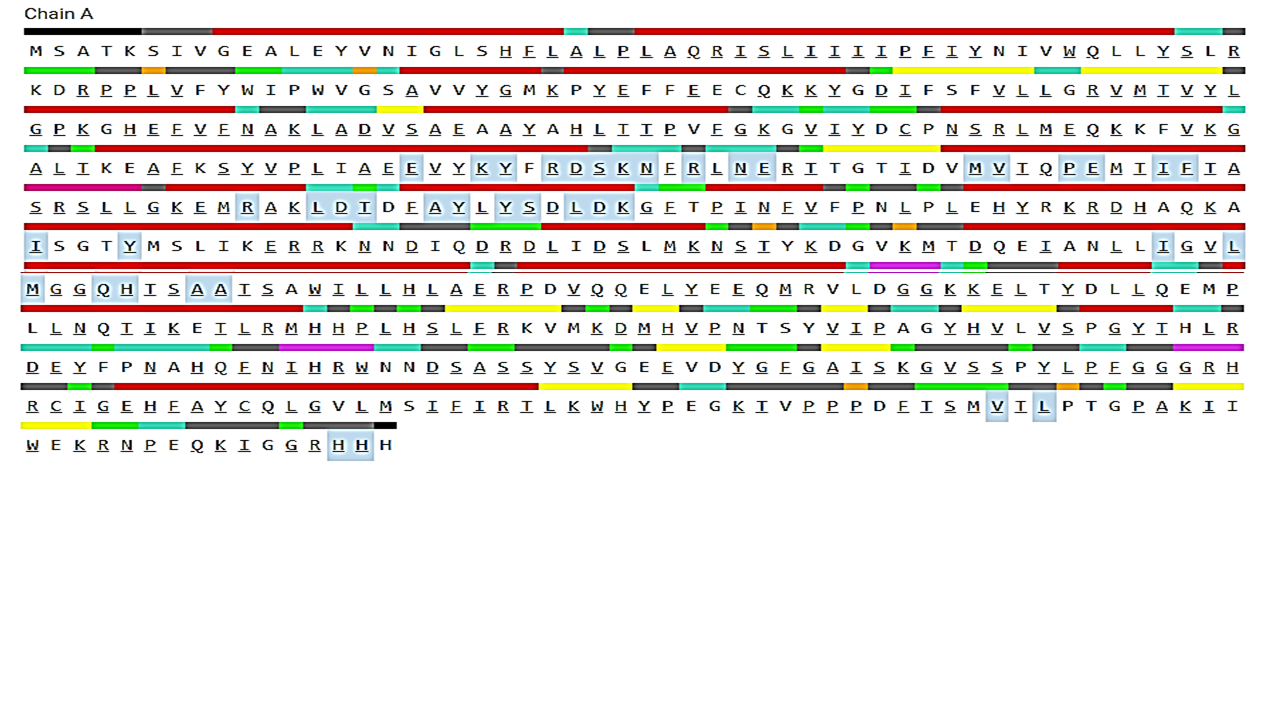


**Figure S3.** Results of amino acids involved in forming active site for Lanosterol 14 - demethylase (CYP51). Letters highlighted in blue indicates active site residues.


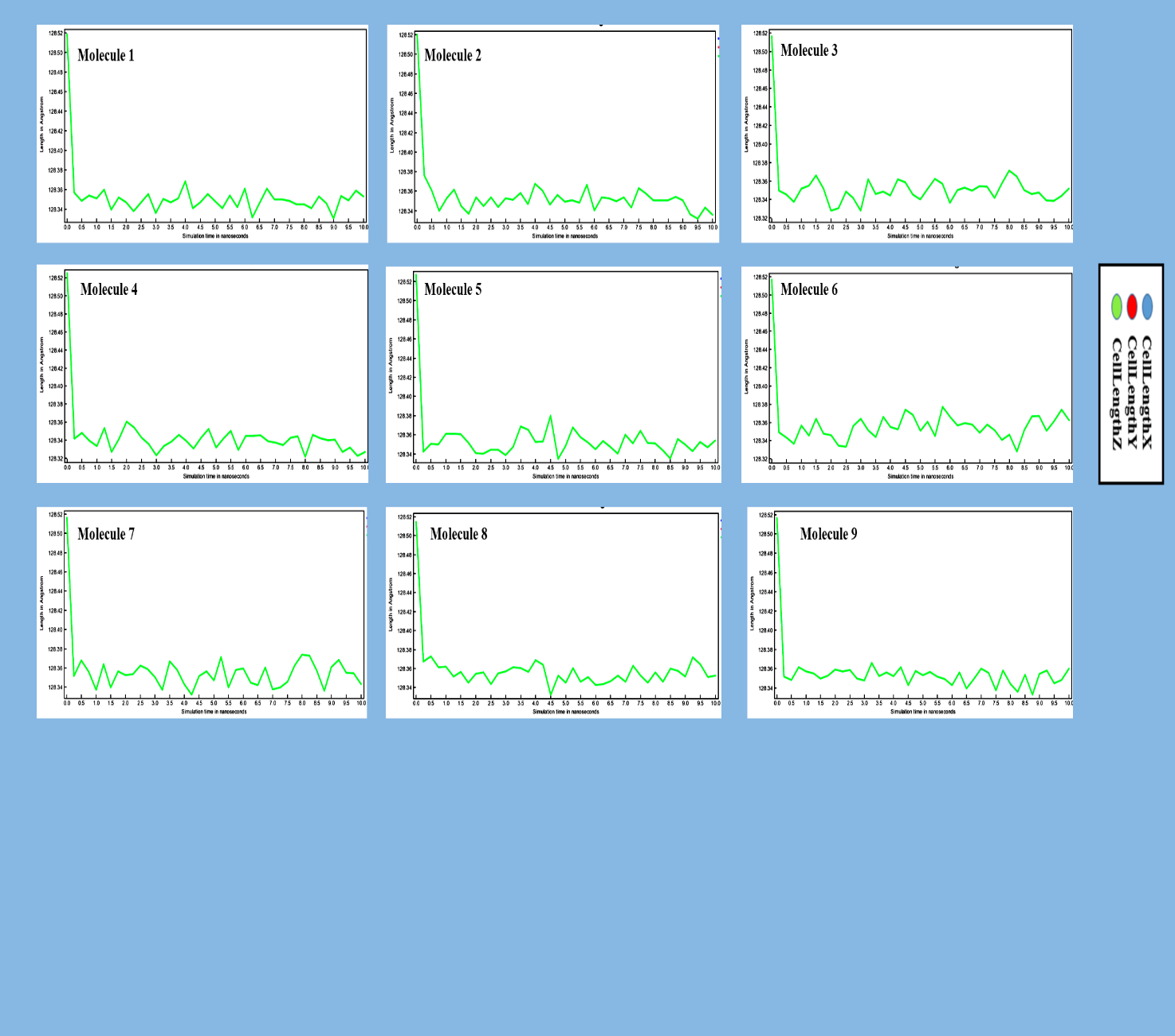


**Figure S4.** Simulation cell lengths calculations of Lanosterol 14 - demethylase (CYP51) and nine molecules as a function of 10ns simulation time.


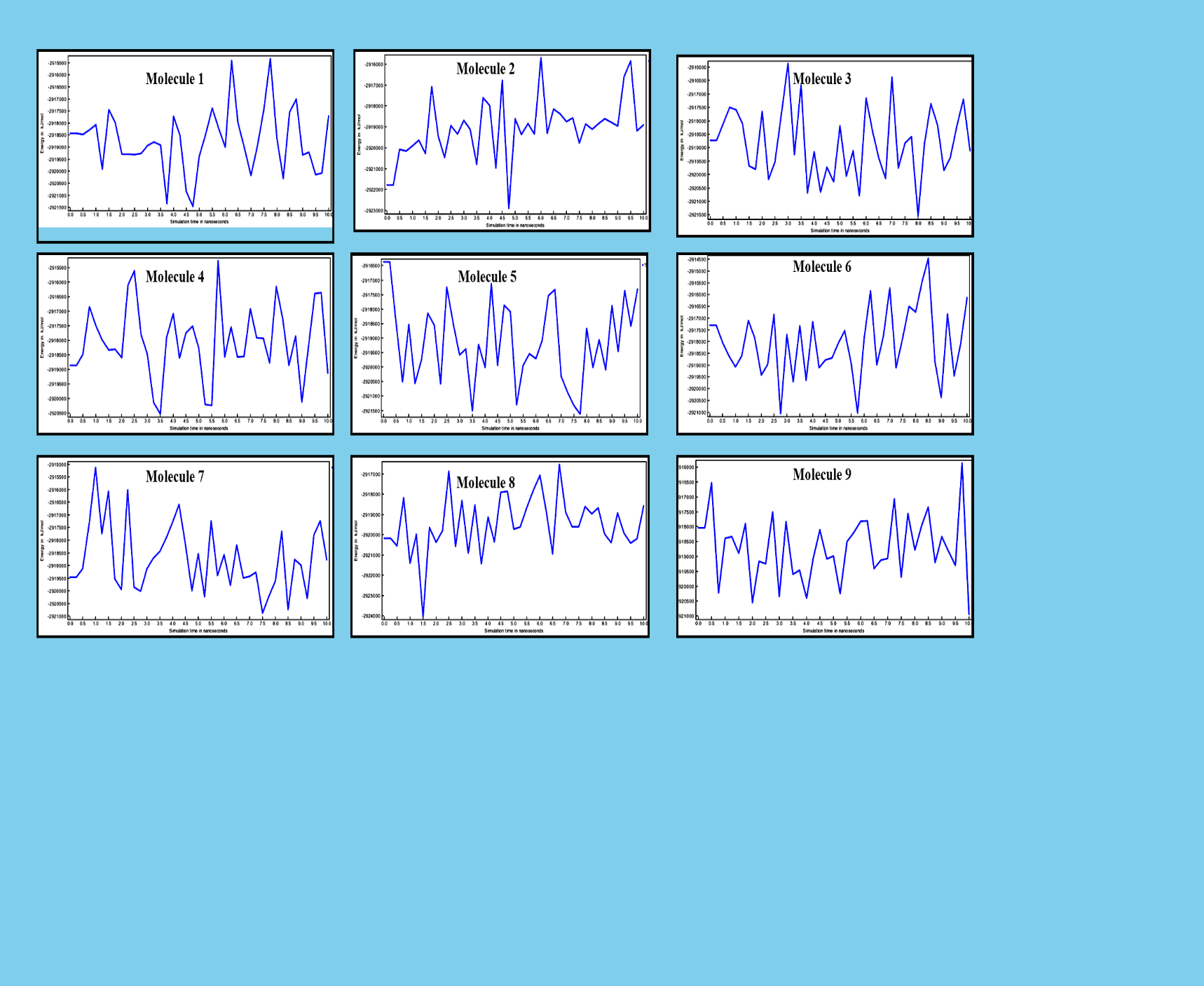


**Figure S5.** Total Potential Energy calculations of Lanosterol 14 - demethylase (CYP51) and nine molecules as a function of 10ns simulation time.


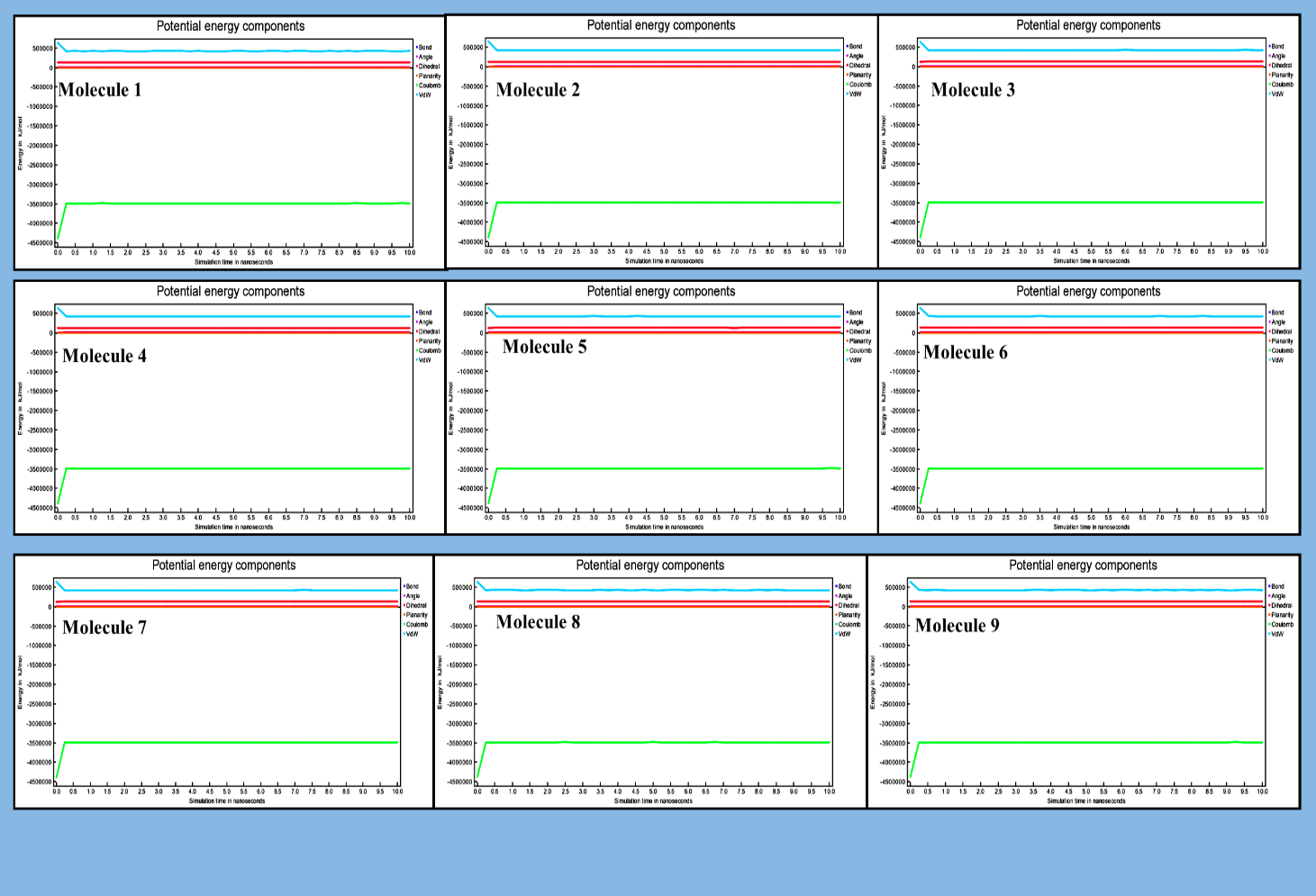


**Figure S6.** Potential Energy calculations of Lanosterol 14 - demethylase (CYP51) and nine molecules as a function of 10ns simulation time.


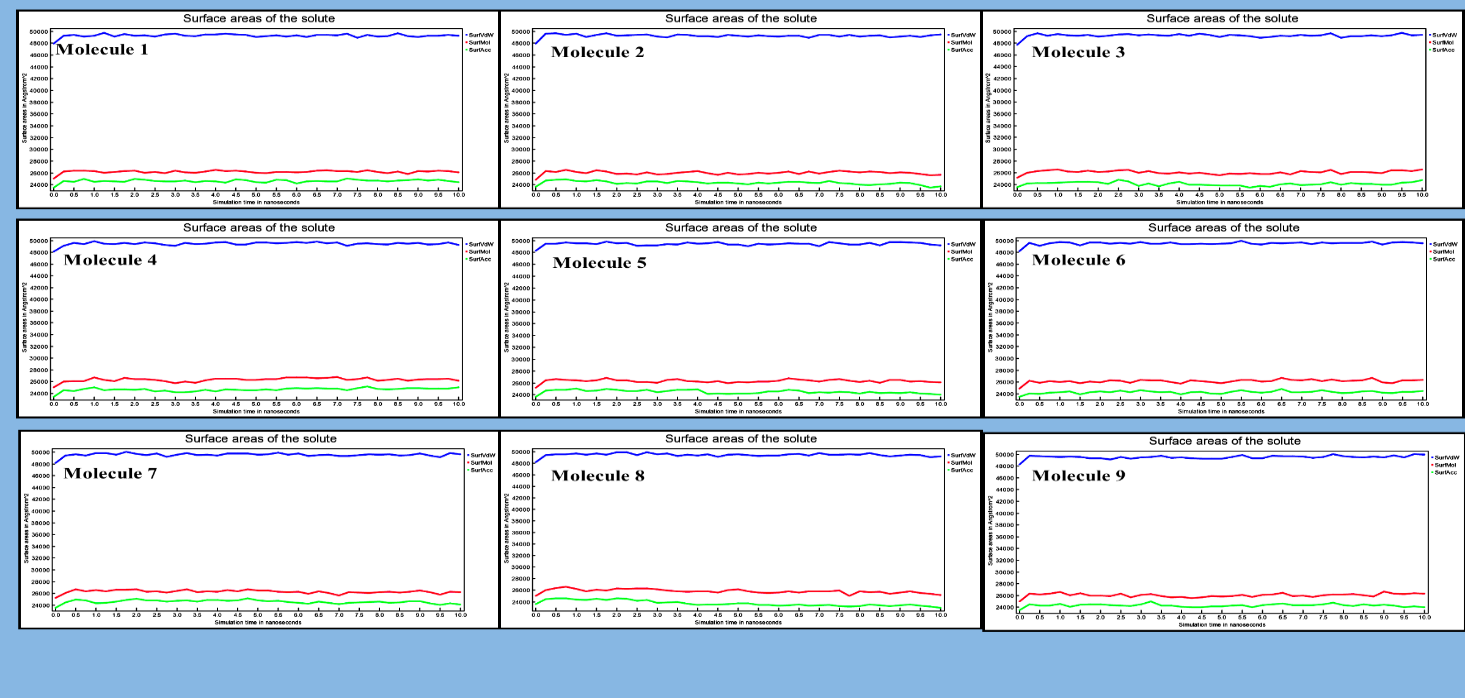


**Figure S7.** Calculations of surface area of the solute of Lanosterol 14 - demethylase (CYP51) and nine molecules as a function of 10ns simulation time.


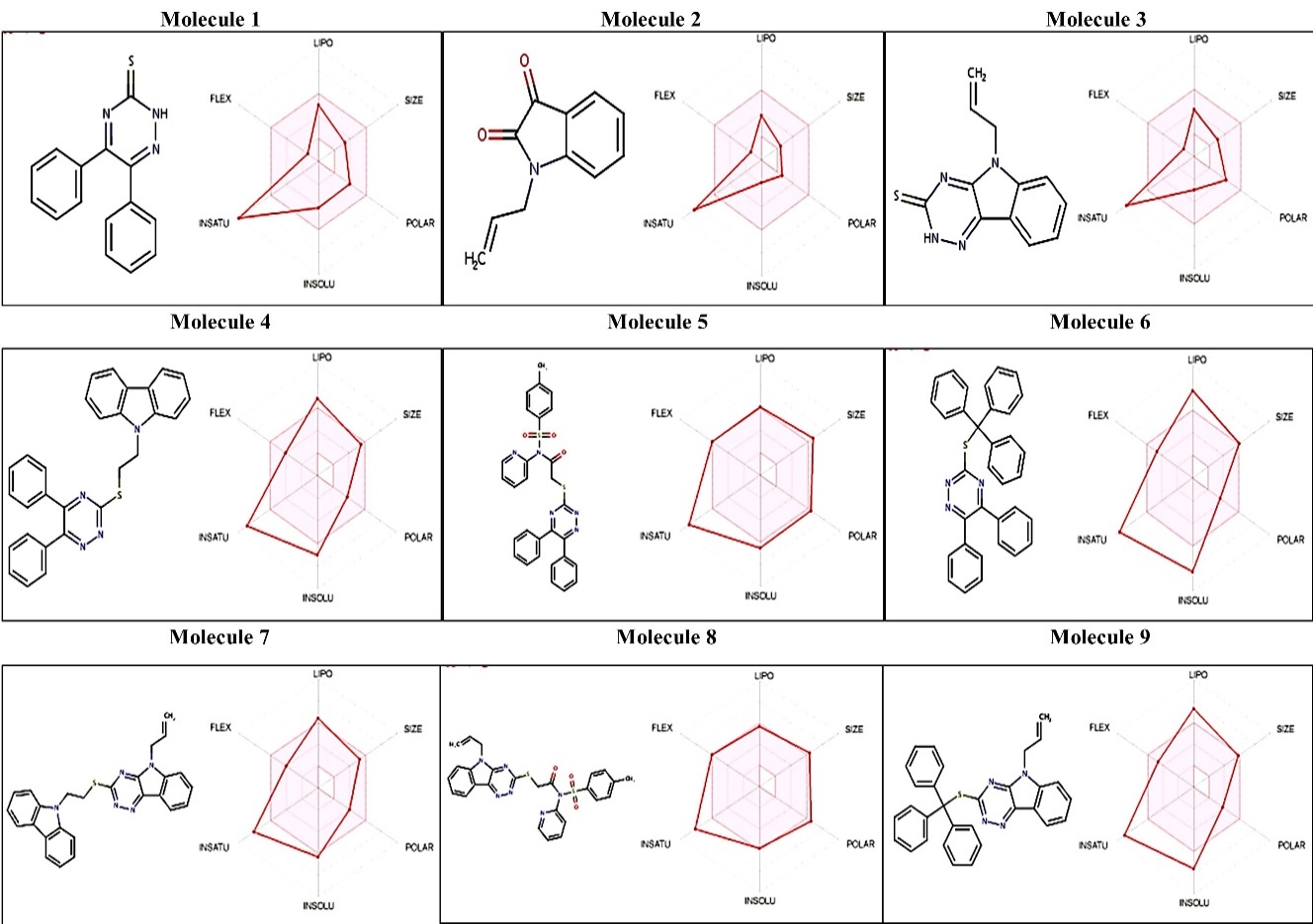


**Figure S8-** All the nine compounds show in a coloured zone are suitable physiochemical space for oral bioavailability and show the LIPO (Lipophilicity), SIZE (Molecular Weight), POLAR (Polarity), INSOLU (Insolubility), INSATU (Instauration), and FLIX (Rotatable bond flexibility) parameters.


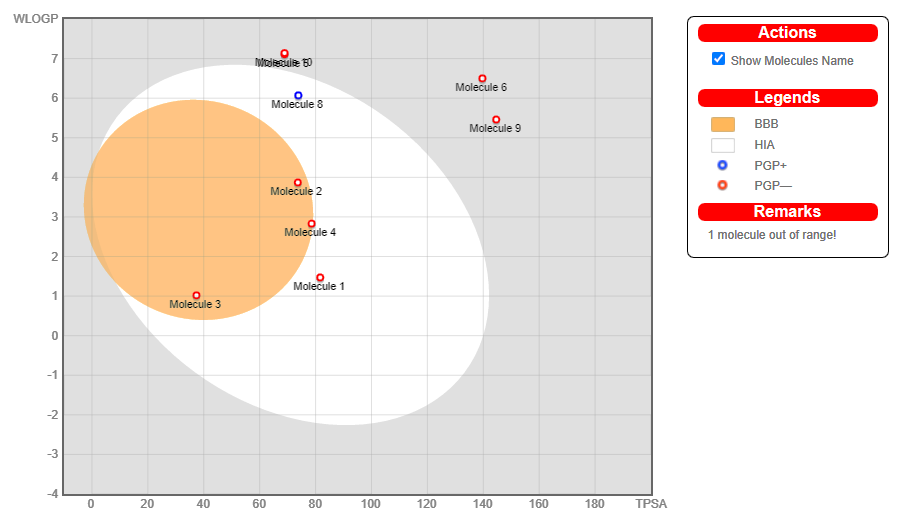


**Figure S9-** Boiled egg diagram and bioavailability radar map of nine compounds and control. (In Figure depicts molecule 1 is control and molecule 2 to 10 is compounds).

**Table S1.** List of amino acids in the active site pocket of Lanosterol 14 - demethylase (CYP51) obtained from CASTp Web server.

| **Targeted Proteins** | **Chain** | **Amino Acids Present In Active Site Pockets** |
| --- | --- | --- |
| Lanosterol 14 - demethylase (CYP51) | A Chain | Glu173, Lys176, Tyr177, Arg179, Asp180, Ser181, Lys182, Asn183, Arg185, Asn187, Glu188, Met197, Val198, Pro201, Glu202, Ile205, Phe206, Arg218, Leu221, Asp222, Thr223, Ala226, Tyr227, Tyr229, Ser230, Leu232, Asp233, Lys234, Ile261, Tyr265, Ile309, Leu312, Met313, Gln316, His317, Ala320, Ala321, Val510, Leu512, His534, His535 |

**Table S2.** Protein-Ligand interactions profile of the Lanosterol 14 - demethylase (CYP51) (PDB id- 4LXJ) with nine molecules.

| **N^0^ Compounds** | **Molecule Name** | **Conventional Hydrogen Bond Interactions with distance** | **Carbon Hydrogen Interactions with distance** | **π –σ Interactions with distance** | **π -Alkyl & Alkyl interactions**  **with distance** | **π- π Stacked/T-Shaped Interactions with distance** | **Pi-Cation and Anion interactions with distance** |
| --- | --- | --- | --- | --- | --- | --- | --- |
| 1. | 1,2,4-Triazine | Thr A: 507 (4.47) | **-** | - | Leu A:95 (5.92), Pro A:238 (5.06), Pro A:238 (5.04) | Phe A:384 (7.09), Phe A:241 (4.35), Met A:509 (6.15) | **-** |
| 2. | 1-(prop-2-en-1-yl)-1*H*-indole-2,3-dione | Arg A:98 (5.12) | - | - | His A:381 (5.88), Leu A:95 (6.15), Tyr A:72 (5.48) | Phe A:384 (6.66 & 6.27) | Phe A:241 |
| 3. | 5-(prop-2-en-1-yl)-5*H*-[1,2,4]triazino[5,6-*b*]indole-3-thiol | Thr A:507 (3.88), Phe A:506 (6.91 & 6.45), Ser A:508 (4.43), Met A:509 (4.40) | **-** | **-** | Pro A:238 (5.01), Leu A:95 (5.90), Phe A:241 (4.67), Phe A:384 (4.98) | Tyr A:72 (6.35) | His A:381 |
| 4. | 2-((5,6-diphenyl-1,2,4-triazin-3-yl)thio)-N-(pyridin-2-yl)-N-tosylacetamide | **-** | **-** | Leu A:96 (5.32), Val A:242 (5.65) | Leu A:95 (5.91), Ala A:69 (6.57), Val A:66 (6.57), Ile A:239 (6.70), Pro A:238 (4.98 & 5.19) | Phe A:384 (6.96), Phe A:241 (4.28) | His A:381 (6.62) |
| 5. | 2-(2-((5,6-diphenyl-1,2,4-triazin-3-yl)thio)ethoxy)isoindoline-1,3-dione | **-** | **-** | - | Pro A:238 (4.64), Ile A:239 (5.16), Val A:242 (4.58), Leu A:96 (5.99), Leu A:95 (5.47) | Phe A:384 (6.68), Phe A:241 (4.45) | **-** |
| 6. | 5,6-diphenyl-3-(tritylthio)-1,2,4-triazine | **-** | **-** | Gly A:314 (4.21), Leu A:380 (6.47 &5.15) | Val A:311 (5.84), Val A:510 (6.87), Met A:509 (6.87), Leu A:147 (7.23), Ile A:139 (5.96) | Phe A:236 (6.95), Tyr A:126 (5.34), Tyr A:140 (6.73) | **-** |
| 7. | 3-((2-(9H-carbazol-9-yl)ethyl)thio)-5-allyl-5H-[1,2,4]triazino[5,6-b]indole | His A:468 (5.22) | **-** | Leu A:380 (5.04) | Ile A:139 (5.97), Val A:311 (6.17), Tyr A:140 (6.04), Phe A:134 (7.01) | Tyr A:126 (6.81) | **-** |
| 8. | 2-((5-allyl-5*H*-[1,2,4]triazino[5,6-*b*]indol-3-yl)thio)-*N*-(pyridin-2-yl)-*N*-tosylacetamide | **-** | Gly A:314 (3.88), Val A:311 (5.06) | **-** | Phe A:134 (5.21), Ile A:139 (4.27), Ile A:471 (5.07), Leu A:158 (6.46), Tyr A:140 (6.32) | Tyr A:126 (5.01) |  |
| 9. | 5-allyl-3-(tritylthio)-5H-[1,2,4]triazino[5,6-b]indole | **-** | **-** | Leu A:380 (5.35) | Val A:311 (5.33), Leu A:147 (4.72), Lys A:151 (4.84), His A:468 (3.59), Leu A:383 (6.17), Leu A:380 (5.97 & 5.67) | Tyr A:126 (5.14) | **-** |

**Table S3-** MM-PBSA energy (KJol/mol calculation of Lanosterol 14-𝛼-demethylase (CYP51) with nine molecules during 10ns simulation.

| **Time**  **[ns]** | **Molecule**  **1** | **Molecule 2** | **Molecule 3** | **Molecule 4** | **Molecule 5** | **Molecule 6** | **Molecule 7** | **Molecule 8** | **Molecule 9** |
| --- | --- | --- | --- | --- | --- | --- | --- | --- | --- |
| 0 | 6.32 | -8.9 | 7.339 | 108.172 | 52.378 | 94.888 | 56.124 | 102.349 | 42.113 |
| 0.25 | 52.83 | 7.5 | -8.518 | 146.774 | -55.506 | 60.119 | 134.039 | 81.975 | 70.492 |
| 0.5 | 17.454 | 25 | 72.595 | 137.413 | 14.459 | 97.198 | 98.548 | -29.978 | 38.578 |
| 0.75 | 46.329 | 17 | 68.359 | 87.228 | -58.203 | 91.99 | 161.262 | 31.712 | 153.366 |
| 1 | 54.085 | 53 | 69.185 | 169.103 | -63.189 | 103.739 | 137.772 | 24.821 | 60.289 |
| 1.25 | 47.946 | 43 | 60.874 | 148.654 | -75.265 | 105.746 | 174.819 | 0.973 | 132.332 |
| 1.5 | 61.502 | 47 | 52.417 | 147.209 | -59.148 | 54.47 | 92.573 | 66.516 | 84.676 |
| 1.75 | 31.875 | 62 | 62.29 | 113.544 | -25.85 | 115.214 | 127.909 | 21.72 | 132.219 |
| 2 | 51.341 | 69 | 85.906 | 134.098 | -71.062 | 76.155 | 73.004 | -1.788 | 77.132 |
| 2.25 | 52.229 | 57 | 63.732 | 108.764 | -66.067 | 78.55 | 88.214 | 17.474 | 80.936 |
| 2.5 | 79.757 | 75 | 74.328 | 122.45 | -25.457 | 91.593 | 88.164 | 0.619 | 120.108 |
| 2.75 | 110.242 | 45 | 46.718 | 169.503 | -46.326 | 136.549 | 75.447 | 2.416 | 128.62 |
| 3 | 61.103 | 69 | 72.429 | 137.151 | -3.832 | 89.908 | 59.781 | -10.154 | 81.753 |
| 3.25 | 73.602 | 91 | 69.88 | 178.746 | 66.506 | 112.934 | 74.112 | 3.227 | 132.391 |
| 3.5 | 81.433 | 57 | 78.716 | 112.508 | 56.898 | 119.365 | 114.676 | 53.104 | 97.732 |
| 3.75 | 71.09 | 77 | 75.523 | 121.01 | 38.84 | 82.517 | 131.789 | 68.259 | 121.277 |
| 4 | 74.04 | 73 | 89.419 | 55.598 | 35.979 | 80.204 | 146.472 | 76.138 | 156.832 |
| 4.25 | 51.682 | 83 | 88.533 | 132.632 | 9.5 | 81.93 | 155.13 | 56.284 | 146.866 |
| 4.5 | 81.591 | 71 | 83.159 | 98.949 | 33.931 | 82.871 | 164.902 | 78.189 | 146.282 |
| 4.75 | 73.438 | 62 | 84.582 | 169.28 | 75.344 | 116.975 | 130.535 | 56.502 | 123.461 |
| 5 | 56.252 | 64 | 102.004 | 73.864 | 109.219 | 87.3 | 130.32 | 80.317 | 166.293 |
| 5.25 | 85.554 | 62 | 80.804 | 163.478 | 82.272 | 86.781 | 131.764 | 91.28 | 159.71 |
| 5.5 | 82.463 | 59 | 94.725 | 146.977 | 89.977 | 67.54 | 126.637 | 86.379 | 137.845 |
| 5.75 | 66.696 | 73 | 58.284 | 149.142 | 83.025 | 112.287 | 122.82 | 93.662 | 110.309 |
| 6 | 84.404 | 72 | 42.936 | 117.117 | 95.969 | 40.046 | 106.355 | 62.809 | 135.78 |
| 6.25 | 84.88 | 76 | 28.908 | 139.494 | 65.656 | 78.283 | 100.468 | 111.939 | 132.744 |
| 6.5 | 58.446 | 85 | 55.202 | 164.207 | 84.818 | 71.067 | 127.531 | 118.344 | 104.934 |
| 6.75 | 66.502 | 62 | 71.738 | 136.38 | 63.52 | 70.126 | 98.222 | 62.139 | 143.947 |
| 7 | 104.781 | 57 | 71.834 | 144.576 | 29.917 | 72.863 | 98.988 | 119.018 | 96.082 |
| 7.25 | 80.109 | 65 | 56.225 | 147.806 | 84.215 | 127.26 | 104.533 | 129.999 | 197.473 |
| 7.5 | 107.14 | 36 | 60.884 | 108.94 | 80.36 | 180.88 | 138.359 | 116.699 | 140.982 |
| 7.75 | 65.784 | 49 | 52.507 | 152.314 | 72.582 | 81.73 | 140.704 | 112.062 | 130.318 |
| 8 | 67.225 | 76 | 62.769 | 137.859 | 90.355 | 115.929 | 141.347 | 136.34 | 146.719 |
| 8.25 | 69.908 | 69 | 55.626 | 162.634 | 76.623 | 96.203 | 125.205 | 147.145 | 177.93 |
| 8.5 | 82.342 | 93 | 79.961 | 134.241 | 47.865 | 137.018 | 153.372 | 125.397 | 161.814 |
| 8.75 | 88.764 | 71 | 73.124 | 94.082 | 44.999 | 126.188 | 146.157 | 129.887 | 133.132 |
| 9 | 94.004 | 68 | 79.891 | 126.085 | 31.726 | 123.476 | 143.899 | 89.9 | 89.297 |
| 9.25 | 92.754 | 71 | 66.273 | 117.783 | -60.505 | 136.42 | 181.276 | 120.774 | 120.313 |
| 9.5 | 77.342 | 72 | 72.63 | 110.343 | -7.103 | 92.475 | 92.652 | 115.065 | 161.726 |
| 9.75 | 98.894 | 74 | 71.744 | 122.132 | -24.946 | 106.815 | 67.761 | 89.948 | 125.751 |
| 10 | 97.742 | 85 | 49.313 | 118.622 | -4.55 | 127.444 | 144.27 | 112.634 | 174.726 |

**Table S4–** Biological activity prediction of the compounds.

| **Molecules** | **Name** | **Pa** | **Pi** | **Biological Activity** |  |
| --- | --- | --- | --- | --- | --- |
|  | 1,2,4-Triazine | 0.296 | 0.082 | Antifungal |  |
|  | 1-(prop-2-en-1-yl)-1*H*-indole-2,3-dione | 0.290 | 0.085 | Antifungal |  |
|  | 5-(prop-2-en-1-yl)-5*H*-[1,2,4] triazino [5,6-*b*]indole-3-thiol | 0.240 | 0.112 | Antifungal |  |
| **Pa = probability to be active; Pi = probability to be inactive** | | | | | |
